# Abortion Surveillance — United States, 2019

**DOI:** 10.15585/mmwr.ss7009a1

**Published:** 2021-11-26

**Authors:** Katherine Kortsmit, Michele G. Mandel, Jennifer A. Reeves, Elizabeth Clark, H. Pamela Pagano, Antoinette Nguyen, Emily E. Petersen, Maura K. Whiteman

**Affiliations:** 1 Division of Reproductive Health, National Center for Chronic Disease Prevention and Health Promotion, CDC

## Abstract

**Problem/Condition:**

CDC conducts abortion surveillance to document the number and characteristics of women obtaining legal induced abortions and number of abortion-related deaths in the United States.

**Period Covered:**

2019.

**Description of System:**

Each year, CDC requests abortion data from the central health agencies for 50 states, the District of Columbia, and New York City. For 2019, 49 reporting areas voluntarily provided aggregate abortion data to CDC. Of these, 48 reporting areas provided data each year during 2010–2019. Census and natality data were used to calculate abortion rates (number of abortions per 1,000 women aged 15–44 years) and ratios (number of abortions per 1,000 live births), respectively. Abortion-related deaths from 2018 were assessed as part of CDC’s Pregnancy Mortality Surveillance System (PMSS).

**Results:**

A total of 629,898 abortions for 2019 were reported to CDC from 49 reporting areas. Among 48 reporting areas with data each year during 2010–2019, in 2019, a total of 625,346 abortions were reported, the abortion rate was 11.4 abortions per 1,000 women aged 15–44 years, and the abortion ratio was 195 abortions per 1,000 live births. From 2018 to 2019, the total number of abortions increased 2% (from 614,820 total abortions), the abortion rate increased 0.9% (from 11.3 abortions per 1,000 women aged 15–44 years), and the abortion ratio increased 3% (from 189 abortions per 1,000 live births). From 2010 to 2019, the total number of reported abortions, abortion rate, and abortion ratio decreased 18% (from 762,755), 21% (from 14.4 abortions per 1,000 women aged 15–44 years), and 13% (from 225 abortions per 1,000 live births), respectively.

In 2019, women in their 20s accounted for more than half of abortions (56.9%). Women aged 20–24 and 25–29 years accounted for the highest percentages of abortions (27.6% and 29.3%, respectively) and had the highest abortion rates (19.0 and 18.6 abortions per 1,000 women aged 20–24 and 25–29 years, respectively). By contrast, adolescents aged <15 years and women aged ≥40 years accounted for the lowest percentages of abortions (0.2% and 3.7%, respectively) and had the lowest abortion rates (0.4 and 2.7 abortions per 1,000 women aged <15 and ≥40 years, respectively). However, abortion ratios in 2019 were highest among adolescents (aged ≤19 years) and lowest among women aged 25–39 years. Abortion rates decreased from 2010 to 2019 for all women, regardless of age. The decrease in abortion rate was highest among adolescents compared with any other age group. From 2018 to 2019, abortion rates decreased or did not change among women aged ≤24 years; however, the abortion rate increased among those aged ≥25 years. Abortion ratios also decreased or did not change from 2010 to 2019 for all age groups, except adolescents aged <15 years. The decrease in abortion ratio was highest among women aged ≥40 years compared with any other age group. From 2018 to 2019, abortion ratios increased for all age groups, except adolescents aged <15 years.

In 2019, 79.3% of abortions were performed at ≤9 weeks’ gestation, and nearly all (92.7%) were performed at ≤13 weeks’ gestation. During 2010–2019, the percentage of abortions performed at >13 weeks’ gestation remained consistently low (≤9.0%). In 2019, the highest proportion of abortions were performed by surgical abortion at ≤13 weeks’ gestation (49.0%), followed by early medical abortion at ≤9 weeks’ gestation (42.3%), surgical abortion at >13 weeks’ gestation (7.2%), and medical abortion at >9 weeks’ gestation (1.4%); all other methods were uncommon (<0.1%). Among those that were eligible (≤9 weeks’ gestation), 53.7% of abortions were early medical abortions. In 2018, the most recent year for which PMSS data were reviewed for pregnancy-related deaths, two women died as a result of complications from legal induced abortion.

**Interpretation:**

Among the 48 areas that reported data continuously during 2010–2019, overall decreases were observed during 2010–2019 in the total number, rate, and ratio of reported abortions; however, from 2018 to 2019, 1%–3% increases were observed across all measures.

**Public Health Action:**

Abortion surveillance can be used to help evaluate programs aimed at promoting equitable access to patient-centered quality contraceptive services in the United States to reduce unintended pregnancies.

## Introduction

This report summarizes data on legal induced abortions for 2019 that were provided voluntarily to CDC by the central health agencies of 49 reporting areas (47 states, the District of Columbia, and New York City, excluding California, Maryland, and New Hampshire) and comparisons over time for the 48 reporting areas that reported each year during 2010–2019 (47 states and New York City). This report also summarizes abortion-related deaths reported voluntarily to CDC for 2018 as part of the Pregnancy Mortality Surveillance System (PMSS).

Since 1969, CDC has conducted abortion surveillance to document the number and characteristics of women obtaining legal induced abortions in the United States. After nationwide legalization of abortion in 1973, the total number, rate (number of abortions per 1,000 women aged 15–44 years), and ratio (number of abortions per 1,000 live births) of reported abortions increased rapidly, reaching the highest levels in the 1980s, before decreasing at a slow yet steady pace (*1*,*2*). During 2006–2008, a break occurred in the previously sustained pattern of decrease (*3*,*4*), although this break was followed in subsequent years by even greater decreases (*5*,*6*). In 2017, the total number, rate, and ratio of reported abortions reached historic lows, followed by 1%–2% increases across all measures from 2017 to 2018 (*5*). Nonetheless, despite the overall decreases, abortion incidence and practices have varied over the years and continue to vary across subpopulations (*7*–*11*), highlighting the need for continued surveillance.

## Methods

### Description of the Surveillance System

Each year, CDC requests aggregated data from the central health agencies of the 50 states, the District of Columbia, and New York City to document the number and characteristics of women obtaining legal induced abortions in the United States. Not all persons who obtain abortions identify as women; the term “women” has been maintained in this report to be consistent with the collection and reporting of denominator data used to calculate abortion rates and ratios. This report contains data voluntarily reported to CDC as of April 9, 2021. For the purpose of surveillance, legal induced abortion is defined as an intervention performed within the limits of state law by a licensed clinician (e.g., a physician, nurse-midwife, nurse practitioner, or physician assistant) intended to terminate a suspected or known intrauterine pregnancy and that does not result in a live birth. All abortions in this report are considered to be legally induced unless stated otherwise.

In most states and jurisdictions, collection of abortion data is facilitated by a legal requirement for hospitals, facilities, or physicians to report abortions to a central health agency (*12*); however, reporting is not complete in all areas, including in some areas with reporting requirements (*13*). Because the reporting of abortion data to CDC is voluntary, many reporting areas have developed their own data collection forms and might not collect or provide all the information requested by CDC. As a result, the level of detail reported by CDC on the characteristics of women obtaining abortions might vary from year to year and by reporting area. To encourage uniform collection of data, CDC has collaborated with the National Association for Public Health Statistics and Information Systems to develop reporting standards and provide technical guidance for vital statistics personnel who collect and summarize abortion data within the United States.

### Variables and Categorization of Data

Each year, CDC sends a suggested template to central health agencies in the United States for compilation of aggregated abortion data among women obtaining legal induced abortions. Aggregate abortion numbers, without individual-level records, are requested for the following variables:

- Age group in years of women obtaining legal induced abortions (<15, 15–19 [age group and by individual year], 20–24, 25–29, 30–34, 35–39, or ≥40)
- Gestational age of pregnancy in completed weeks at the time of abortion (≤6, 7–20 by individual week, or ≥21)
- Race (Black, White, or other [including Asian, Pacific Islander, other races, and multiple races]), ethnicity (Hispanic or non-Hispanic), and race by ethnicity
- Method type (surgical abortion, intrauterine instillation, medical [nonsurgical] abortion, or hysterectomy/hysterotomy)
- Marital status (married [including currently married or separated] or unmarried [including never married, widowed, or divorced])
- Number of previous live births (zero, one, two, three, or four or more)
- Number of previous induced abortions (zero, one, two, or three or more)
- Residence (the state, jurisdiction, territory, or foreign country in which the women obtaining the abortion lived, or, if additional details are unavailable, in-reporting area versus out-of-reporting area)

In addition, the template provided by CDC requests that aggregate numbers for certain variables be cross-tabulated by a second variable. The cross-tabulations presented in this report include weeks of gestation separately by method type, by age group, and by race/ethnicity.

Beginning with 2014 data, instead of reporting the clinicians’ estimates of gestational age or estimates of gestational age based on last menstrual period, some areas have reported “probable postfertilization age,” “clinician’s estimate of gestation based on date of conception,” and “probable gestational age” to CDC. To ensure consistency between data reported as postfertilization age and the data collection practices for gestational age recommended by CDC’s National Center for Health Statistics (*14*), 2 weeks were added to probable postfertilization age. This method was used to account for time after last menstrual period until ovulation in a standard 28-day cycle because fertilization occurs around the time of ovulation (*15*). No modifications were made to data reported as clinician’s estimate of gestational age based on date of conception or data reported as probable gestational age.

In this report, medical and surgical abortions are further categorized by gestational age when available in the categories reported by CDC. Early medical abortion is defined as the administration of medications (typically mifepristone followed by misoprostol) to induce an abortion at ≤9 completed weeks’ gestation consistent with the current Food and Drug Administration (FDA) labeling for mifepristone (implemented in 2016) (*16*). CDC collects information only on the estimated number of weeks (not days) of gestation and acknowledges the conventional use of completed weeks of gestation to describe pregnancy duration; therefore, CDC’s category of ≤9 weeks’ gestation includes abortions through 9 weeks and 6 days. Medications (typically serial prostaglandins, sometimes administered after mifepristone) may also be used to induce an abortion at >9 weeks’ gestation. Surgical abortions, which include uterine aspiration (i.e., dilation and curettage, aspiration curettage, suction curettage, manual vacuum aspiration, menstrual extraction, or sharp curettage) and dilation and evacuation procedures, are categorized as having been performed at ≤13 weeks’ gestation or at >13 weeks’ gestation because of differences in surgical technique at these gestational ages (*17*). Finally, because intrauterine instillations are unlikely to be performed early in gestation (*18*), abortions reported to have been performed by intrauterine instillation at ≤12 weeks’ gestation are excluded from calculation of the percentage of abortions by known method type and are grouped with unknown type. The cutoff of ≤12 weeks was selected because this procedure is unlikely to be performed at earlier gestational ages.

### Measures of Abortion

Four measures of abortion are presented in this report: 1) the number of abortions in a given population, 2) the percentage of abortions among women by selected characteristics, 3) the abortion rate (number of abortions per 1,000 women within a given population), and 4) the abortion ratio (number of abortions per 1,000 live births within a given population). Abortion rates adjust for differences in population size. Abortion ratios measure the relative number of pregnancies in a population that end in abortion compared with live birth.

The U.S. Census Bureau estimates of the resident female population were used as the denominator for calculating abortion rates (*19*–*28*). Overall abortion rates were calculated from the population of women aged 15–44 years living in the reporting areas that provided continuously reported data. For adolescents aged <15 years, abortion rates were calculated using the number of adolescents aged 13–14 years; for women aged ≥40 years, abortion rates were calculated using the number of women aged 40–44 years. For the calculation of abortion ratios, live birth data were obtained from CDC natality files and included births to women of all ages living in the reporting areas that provided abortion data (*29**,**30*). For calculation of the total abortion rates and total ratios only, women with unknown data on selected characteristics (e.g., age, race/ethnicity, and marital status) were distributed according to the distribution of abortions among those with known information on the characteristic. For calculation of totals only, abortions for women with an unknown gestational age of pregnancy but known method type were distributed according to the distribution of abortions among those with known information on method type by gestational age to the following categories: surgical, ≤13 weeks’ gestation; surgical, >13 weeks’ gestation; medical ≤9 weeks’ gestation; and medical >9 weeks’ gestation.

### Data Presentation and Analysis

This report provides aggregate and reporting area–specific abortion numbers, rates, and ratios for the 49 areas that reported to CDC for 2019, which excluded California, Maryland, and New Hampshire. In addition, this report describes characteristics of women who obtained abortions in 2019. The data in this report are presented by the reporting area in which the abortions were performed.

The completeness and quality of data received vary by year and by variable; this report only describes the characteristics of women obtaining abortions in reporting areas that met CDC reporting standards (i.e., reported at least 20 abortions overall, provided data categorized in accordance with requested variables, and had <15% unknown values for a given characteristic). Cells with a value in the range of 1–4 or cells that would allow for calculation of these values have been suppressed in this report to maintain confidentiality in tables presented by reporting area of occurrence.

Trends in the number, rate, and ratio of reported abortions and annual data are presented for the 48 areas that reported every year during 2010–2019. The percentage change in abortion measures from the most recent past year (2018 to 2019) and during the 10-year period of analysis (2010 to 2019) were calculated for these 48 reporting areas.

Trends are also reported for abortions by age group, weeks of gestation, and early medical abortions (≤9 completed weeks’ gestation). Annual data are presented for areas that met reporting standards every year during 2010–2019; the percentage change was calculated from the beginning to the end of the 10-year period of analysis (2010–2019), in 5-year increments from the beginning to the end of the first and second halves of this period (2010–2014 and 2015–2019), and from the most recent past year (2018 to 2019). Consistent with previous reports (*5*), key findings for trends are presented to highlight observed changes over time and differences between groups. Trends for early medical abortions are reported to monitor any changes in clinical practice that might have occurred with the accumulation of evidence on the safety and effectiveness of medical abortion past 63 days of gestation (8 completed weeks’ gestation) (*31*), changes in professional practice guidelines published in 2013 and 2014 (*32*,*33*), and the 2016 FDA extension of the gestational age limit for the use of mifepristone for early medical abortion from 63 days to 70 days (9 completed weeks’ gestation) (*34*). No statistical testing was performed. Comparisons do not imply statistical significance, and lack of comment regarding the difference between values does not imply that no statistically significant difference exists.

Data from reporting areas are not included in trends if the data did not meet reporting standards every year during 2010–2019. As a result, aggregate measures for 2019 in trend analyses might differ from the point estimates reported for 2019.

### Abortion Mortality

CDC has reported data on abortion-related deaths periodically since information on abortion mortality first was included in the 1972 abortion surveillance report (*5*,*35*). An abortion-related death is defined as a death resulting from a direct complication of an abortion (legal or illegal), an indirect complication caused by a chain of events initiated by an abortion, or an aggravation of a preexisting condition by the physiologic or psychologic effects of abortion (*36*). An abortion is categorized as legal when it is performed by a licensed clinician within the limits of state law.

Since 1987, CDC has monitored abortion-related deaths through PMSS (*37*). Sources of data to identify abortion-related deaths have included state vital records; media reports, including computerized searches of full-text newspaper and other print media databases; and individual case reports by public health agencies, including maternal mortality review committees, health care providers and provider organizations, private citizens, and citizen groups. For each death that is possibly related to abortion, CDC requests clinical records and autopsy reports. Two medical epidemiologists independently review these reports to determine the cause of death and whether the death was abortion related. Discrepancies are discussed and resolved by consensus. Each death is categorized by abortion type as legal induced, illegal induced, spontaneous, or unknown type.

This report provides PMSS data on induced abortion-related deaths that occurred in 2018, the most recent year for which PMSS data are available. Data on induced abortion-related deaths that occurred during 1972–2017 have been published (*1*,*5*,*38*). For 1998–2018, abortion surveillance data reported to CDC cannot be used alone to calculate national case-fatality rates for legal induced abortions (number of legal induced abortion-related deaths per 100,000 reported legal induced abortions in the United States) because eight reporting areas did not report abortion data every year during this period (Alaska, 1998–2000; California, 1998–2018; District of Columbia, 2016; Louisiana, 2005; Maryland, 2007–2018; New Hampshire, 1998–2018; Oklahoma, 1998–1999; and West Virginia, 2003–2004). Thus, denominator data for calculation of national legal induced abortion case-fatality rates were obtained from a published report by the Guttmacher Institute that includes estimated total numbers of abortions in the United States from a national survey of abortion-providing facilities (*6*). For 2018, the case-fatality rate was calculated using denominator data for 2017, the most recent year for which data from the Guttmacher Institute are available. Because rates determined on the basis of a numerator of <20 deaths are unstable (*39*), national case-fatality rates for legal induced abortion were calculated for consecutive 5-year periods during 1973–2012 and then for a consecutive 6-year period during 2013–2018.

## Results

### Total Abortions Reported to CDC by Occurrence

Among the 49 reporting areas that provided data for 2019, a total of 629,898 abortions were reported. Of these abortions, 625,346 (99.3%) were from 48 reporting areas that provided data every year during 2010–2019. In 2019, these continuously reporting areas had an abortion rate of 11.4 abortions per 1,000 women aged 15–44 years and an abortion ratio of 195 abortions per 1,000 live births (Table 1). From 2018 to 2019, the total number of reported abortions increased 2% (from 614,820 total abortions), the abortion rate increased 0.9% (from 11.3 abortions per 1,000 women aged 15–44 years), and the abortion ratio increased 3% (from 189 abortions per 1,000 live births). From 2010 to 2019, the total number of reported abortions decreased 18% (from 762,755), the abortion rate decreased 21% (from 14.4 abortions per 1,000 women aged 15–44 years), and the abortion ratio decreased 13% (from 225 abortions per 1,000 live births) (Figure).

**TABLE 1:** Number, percentage, rate,* and ratio^†^ of reported abortions — selected reporting areas, United States, 2010–2019

Year	Selected reporting areas^§^	Continuously reporting areas^¶^		
	No.	No. (%)**	Rate	Ratio
2010	765,651	762,755 (99.6)	14.4	225
2011	730,322	727,554 (99.6)	13.7	217
2012	699,202	696,587 (99.6)	13.1	208
2013	664,435	661,874 (99.6)	12.4	198
2014	652,639	649,849 (99.6)	12.1	192
2015	638,169	636,902 (99.8)	11.8	188
2016	623,471	623,471 (100.0)	11.6	186
2017	612,719	609,095 (99.4)	11.2	185
2018	619,591	614,820 (99.2)	11.3	189
2019	629,898	625,346 (99.3)	11.4	195

* Number of abortions per 1,000 women aged 15–44 years.

^†^ Number of abortions per 1,000 live births.

^§^ For each given year, excludes reporting areas that did not report that year’s abortion numbers to CDC: California (2010–2019), District of Columbia (2016), Maryland (2010–2019), and New Hampshire (2010–2019).

^¶^ For all years, excludes reporting areas that did not report abortion numbers every year during the period of analysis (2010–2019): California, District of Columbia, Maryland, and New Hampshire.

** Abortions from areas that reported every year during 2010–2019 as a percentage of all reported abortions for a given year.

**FIGURE:**
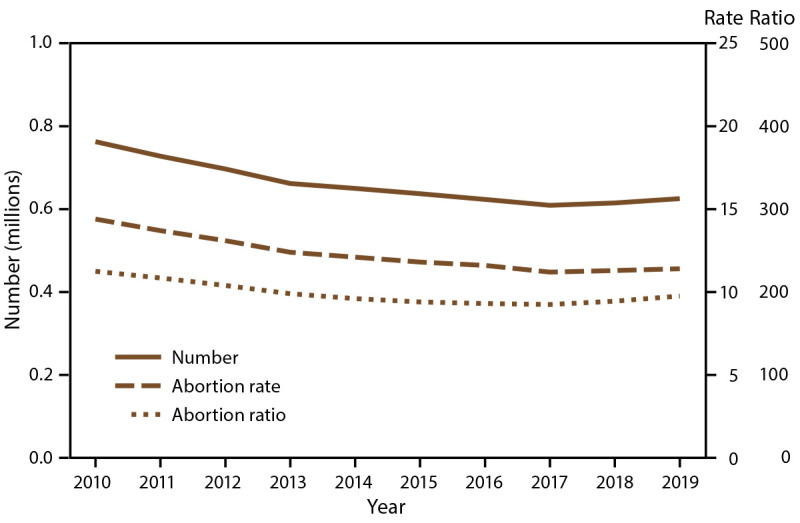
Number, rate,* and ratio^†^ of abortions performed, by year — selected reporting areas,^§^ United States, 2010–2019 * Number of abortions per 1,000 women aged 15–44 years. ^†^ Number of abortions per 1,000 live births. ^§^ Data are for 48 reporting areas; excludes California, District of Columbia, Maryland, and New Hampshire.

In 2019, a considerable range existed in abortion rates by reporting area of occurrence (from 0.3 to 27.2 abortions per 1,000 women aged 15–44 years in Wyoming and New York City) and abortion ratios (from 5 to 501 abortions per 1,000 live births in Wyoming and the District of Columbia) (Table 2). The percentage of abortions obtained by out-of-state residents also varied among reporting areas (from 0.5% in Arizona to 68.7% in the District of Columbia). Overall, 0.9% of abortions were reported to CDC with unknown residence.

**TABLE 2:** Number, rate,* and ratio^†^ of reported abortions, by reporting area of occurrence and number of abortions obtained by out-of-state residents^§^ — United States, 2019^¶^

State/Area	Abortions reported by area of occurrence**			Abortions obtained by out-of-state residents
	No.	Rate	Ratio	No. (%)
Alabama	6,009	6.3	103	1,040 (17.3)
Alaska	1,270	8.8	129	19 (1.5)
Arizona	13,097	9.4	165	67 (0.5)
Arkansas	2,963	5.1	81	338 (11.4)
Colorado	9,002	7.6	143	946 (10.5)
Connecticut	9,202	13.7	269	334 (3.6)
Delaware	2,042	11.3	193	277 (13.6)
District of Columbia	4,552	23.9	501	3,126 (68.7)
Florida	71,914	18.5	327	2,256 (3.1)
Georgia	36,907	16.9	292	6,500 (17.6)
Hawaii	2,003	7.6	119	49 (2.4)
Idaho	1,513	4.4	69	78 (5.2)
Illinois	46,517	18.6	332	7,534 (16.2)
Indiana	7,637	5.8	94	618 (8.1)
Iowa	3,566	6.0	95	490 (13.7)
Kansas	6,894	12.3	195	3,372 (48.9)
Kentucky	3,664	4.3	69	643 (17.5)
Louisiana	8,144	8.8	138	1,358 (16.7)
Maine	2,021	8.7	172	107 (5.3)
Massachusetts	18,593	13.3	269	631 (3.4)
Michigan	27,339	14.6	253	1,435 (5.2)
Minnesota	9,940	9.2	151	888 (8.9)
Mississippi	3,194	5.5	87	335 (10.5)
Missouri	1,471	1.2	20	128 (8.7)
Montana	1,568	8.0	142	169 (10.8)
Nebraska	2,068	5.5	84	267 (12.9)
Nevada	8,414	14.0	240	434 (5.2)
New Jersey^††^	22,178	13.2	223	1,309 (5.9)
New Mexico	3,942	9.9	172	939 (23.8)
New York	78,587	20.3	355	6,989 (8.9)
New York City	49,784	27.2	472	4,668 (9.4)
New York State	28,803	14.1	248	2,321 (8.1)
North Carolina	28,450	13.8	240	5,079 (17.9)
North Dakota	1,121	7.6	107	289 (25.8)
Ohio	20,102	9.1	150	1,186 (5.9)
Oklahoma	4,995	6.4	102	407 (8.1)
Oregon	8,688	10.5	208	795 (9.2)
Pennsylvania	31,018	13.0	231	2,222 (7.2)
Rhode Island	2,099	10.1	206	274 (13.1)
South Carolina	5,101	5.2	89	312 (6.1)
South Dakota	414	2.6	36	82 (19.8)
Tennessee	9,719	7.3	121	1,823 (18.8)
Texas	57,275	9.5	152	1,303 (2.3)
Utah	2,922	4.2	62	146 (5.0)
Vermont	1,195	10.4	223	265 (22.2)
Virginia	15,601	9.2	160	867 (5.6)
Washington	17,262	11.4	203	848 (4.9)
West Virginia	1,183	3.8	65	168 (14.2)
Wisconsin	6,511	6.0	103	139 (2.1)
Wyoming	31	0.3	5	5 (16.1)
**Total**	**629,898**	**NA**	**NA**	**NA**

**Abbreviation:** NA = not applicable.

* Number of abortions per 1,000 women aged 15–44 years.

^†^ Number of abortions per 1,000 live births.

^§^ Additional details on the reporting area in which abortions were provided, cross-tabulated by the state/area of residence, are available at https://www.cdc.gov/reproductivehealth/data_stats/Abortion.htm.

^¶^ Data from 49 reporting areas; excludes three reporting areas (California, Maryland, and New Hampshire) that did not report or did not meet reporting standards.

** The total abortions include those with known and unknown residence status.

^††^ Reporting to the central health agency is not required. Data are requested from hospitals and licensed ambulatory care facilities only.

### Age Group, Race/Ethnicity, and Marital Status

Among the 49 areas that reported abortion numbers by women’s age for 2019, women in their 20s accounted for the majority (56.9%) of abortions (Table 3). Women aged 20–24 and 25–29 years accounted for the highest percentages of abortions (27.6% and 29.3%, respectively) and had the highest abortion rates (19.0 and 18.6 abortions per 1,000 women aged 20–24 and 25–29 years, respectively). By contrast, those in the youngest (<15 years) and oldest (≥40 years) age groups accounted for the smallest percentages of abortions (0.2% and 3.7%) and had the lowest abortion rates (0.4 and 2.7 abortions per 1,000 women aged <15 and ≥40 years). However, abortion ratios in 2019 were highest among adolescents (873 and 348 abortions per 1,000 live births among those aged <15 years and 15–19 years) and lowest among women aged 25–39 years (194, 132, and 145 abortions per 1,000 live births among those aged 25–29, 30–34, and 35–39 years, respectively).

**TABLE 3:** Number of reported abortions, by known age group and reporting area of occurrence — selected reporting areas,* United States, 2019

State/Area	Age group (yrs)							Total abortions reported by known age
	<15	15–19	20–24	25–29	30–34	35–39	≥40	
	No. (%)^†^	No. (%)	No. (%)	No. (%)	No. (%)	No. (%)	No. (%)	No. (% of all reported abortions)^§^
Alabama	23 (0.4)	526 (8.8)	1,807 (30.1)	1,783 (29.7)	1,119 (18.6)	565 (9.4)	183 (3.0)	**6,006 (100.0)**
Alaska	—^¶^	123 (9.7)	351 (27.6)	363 (28.6)	246 (19.4)	137 (10.8)	—^¶^	**1,270 (100.0)**
Arizona	19 (0.1)	1,163 (8.9)	3,932 (30.0)	3,631 (27.7)	2,446 (18.7)	1,420 (10.8)	482 (3.7)	**13,093 (100.0)**
Arkansas	10 (0.3)	294 (9.9)	892 (30.1)	901 (30.4)	513 (17.3)	260 (8.8)	90 (3.0)	**2,960 (99.9)**
Colorado	27 (0.3)	812 (9.0)	2,596 (28.9)	2,557 (28.4)	1,735 (19.3)	948 (10.5)	319 (3.5)	**8,994 (99.9)**
Connecticut	20 (0.2)	788 (8.7)	2,382 (26.3)	2,600 (28.8)	1,885 (20.9)	1,042 (11.5)	323 (3.6)	**9,040 (98.2)**
Delaware	8 (0.4)	240 (11.8)	575 (28.2)	574 (28.1)	388 (19.0)	209 (10.2)	48 (2.4)	**2,042 (100.0)**
District of Columbia	10 (0.2)	386 (8.5)	1,262 (27.7)	1,388 (30.5)	870 (19.1)	483 (10.6)	152 (3.3)	**4,551 (100.0)**
Florida	118 (0.2)	5,231 (7.3)	18,889 (26.5)	20,741 (29.1)	15,051 (21.1)	8,425 (11.8)	2,907 (4.1)	**71,362 (99.2)**
Georgia	71 (0.2)	2,832 (7.7)	10,185 (27.6)	11,361 (30.8)	7,254 (19.7)	3,932 (10.7)	1,272 (3.4)	**36,907 (100.0)**
Hawaii	6 (0.3)	190 (9.5)	549 (27.4)	546 (27.3)	381 (19.0)	238 (11.9)	93 (4.6)	**2,003 (100.0)**
Idaho	—^¶^	187 (12.4)	477 (31.5)	378 (25.0)	252 (16.7)	161 (10.6)	—^¶^	**1,512 (99.9)**
Illinois**	75 (0.2)	3,492 (9.1)	10,960 (28.4)	11,819 (30.6)	7,166 (18.6)	3,813 (9.9)	1,255 (3.3)	**38,580 (99.5)**
Indiana	18 (0.2)	768 (10.1)	2,324 (30.4)	2,168 (28.4)	1,354 (17.7)	745 (9.8)	260 (3.4)	**7,637 (100.0)**
Iowa	12 (0.3)	357 (10.0)	957 (26.8)	1,000 (28.1)	679 (19.0)	401 (11.2)	159 (4.5)	**3,565 (100.0)**
Kansas	12 (0.2)	632 (9.2)	2,148 (31.2)	1,932 (28.0)	1,239 (18.0)	692 (10.0)	239 (3.5)	**6,894 (100.0)**
Kentucky	11 (0.3)	294 (8.0)	1,071 (29.2)	1,069 (29.2)	719 (19.6)	384 (10.5)	116 (3.2)	**3,664 (100.0)**
Louisiana	26 (0.3)	726 (8.9)	2,302 (28.3)	2,521 (31.0)	1,534 (18.8)	782 (9.6)	253 (3.1)	**8,144 (100.0)**
Maine	5 (0.2)	198 (9.8)	585 (29.0)	534 (26.4)	381 (18.9)	254 (12.6)	63 (3.1)	**2,020 (100.0)**
Massachusetts	28 (0.2)	1,306 (7.0)	4,613 (24.8)	5,313 (28.6)	4,028 (21.7)	2,390 (12.9)	910 (4.9)	**18,588 (100.0)**
Michigan	51 (0.2)	2,328 (8.6)	7,538 (27.7)	8,697 (32.0)	5,201 (19.1)	2,545 (9.4)	831 (3.1)	**27,191 (99.5)**
Minnesota	26 (0.3)	823 (8.3)	2,693 (27.1)	2,837 (28.6)	2,015 (20.3)	1,205 (12.1)	336 (3.4)	**9,935 (99.9)**
Mississippi	7 (0.2)	295 (9.2)	970 (30.4)	1,003 (31.4)	574 (18.0)	276 (8.6)	69 (2.2)	**3,194 (100.0)**
Missouri	5 (0.3)	141 (9.6)	422 (28.7)	436 (29.6)	253 (17.2)	156 (10.6)	58 (3.9)	**1,471 (100.0)**
Montana	5 (0.3)	157 (10.0)	458 (29.2)	454 (29.0)	269 (17.2)	161 (10.3)	64 (4.1)	**1,568 (100.0)**
Nebraska	7 (0.3)	187 (9.0)	625 (30.2)	534 (25.8)	400 (19.3)	234 (11.3)	81 (3.9)	**2,068 (100.0)**
Nevada	19 (0.2)	691 (8.6)	2,200 (27.2)	2,257 (27.9)	1,630 (20.2)	918 (11.4)	363 (4.5)	**8,078 (96.0)**
New Jersey^††^	50 (0.2)	1,958 (8.8)	5,648 (25.5)	6,497 (29.3)	4,462 (20.1)	2,604 (11.7)	959 (4.3)	**22,178 (100.0)**
New Mexico	22 (0.6)	507 (13.8)	1,111 (30.3)	921 (25.1)	621 (16.9)	379 (10.3)	104 (2.8)	**3,665 (93.0)**
New York	186 (0.2)	6,919 (8.8)	20,238 (25.8)	22,267 (28.4)	16,014 (20.4)	9,438 (12.0)	3,386 (4.3)	**78,448 (99.8)**
New York City	109 (0.2)	4,052 (8.1)	12,471 (25.1)	14,159 (28.4)	10,414 (20.9)	6,260 (12.6)	2,318 (4.7)	**49,783 (100.0)**
New York State	77 (0.3)	2,867 (10.0)	7,767 (27.1)	8,108 (28.3)	5,600 (19.5)	3,178 (11.1)	1,068 (3.7)	**28,665 (99.5)**
North Carolina	60 (0.2)	2,252 (8.3)	7,768 (28.8)	8,270 (30.6)	5,095 (18.9)	2,687 (9.9)	880 (3.3)	**27,012 (94.9)**
North Dakota	0 (—)	121 (10.8)	349 (31.1)	326 (29.1)	189 (16.9)	107 (9.5)	29 (2.6)	**1,121 (100.0)**
Ohio	63 (0.3)	1,737 (8.6)	5,887 (29.3)	6,057 (30.1)	3,720 (18.5)	1,983 (9.9)	655 (3.3)	**20,102 (100.0)**
Oklahoma	89 (1.8)	440 (8.8)	1,517 (30.4)	1,384 (27.7)	901 (18.0)	491 (9.8)	172 (3.4)	**4,994 (100.0)**
Oregon	19 (0.2)	837 (9.6)	2,373 (27.3)	2,326 (26.8)	1,702 (19.6)	1,049 (12.1)	382 (4.4)	**8,688 (100.0)**
Pennsylvania	71 (0.2)	2,474 (8.0)	8,435 (27.2)	9,529 (30.7)	6,188 (19.9)	3,244 (10.5)	1,077 (3.5)	**31,018 (100.0)**
Rhode Island	—^¶^	185 (8.8)	611 (29.1)	584 (27.8)	411 (19.6)	222 (10.6)	—^¶^	**2,098 (100.0)**
South Carolina	10 (0.2)	499 (9.8)	1,403 (27.5)	1,500 (29.4)	961 (18.8)	544 (10.7)	184 (3.6)	**5,101 (100.0)**
South Dakota	—^¶^	—^¶^	129 (31.2)	117 (28.3)	61 (14.7)	45 (10.9)	—^¶^	**414 (100.0)**
Tennessee	17 (0.2)	830 (8.6)	2,884 (29.8)	2,982 (30.8)	1,791 (18.5)	913 (9.4)	275 (2.8)	**9,692 (99.7)**
Texas	106 (0.2)	5,041 (8.8)	16,647 (29.1)	16,327 (28.5)	10,907 (19.0)	6,152 (10.7)	2,095 (3.7)	**57,275 (100.0)**
Utah	6 (0.2)	371 (12.7)	915 (31.3)	761 (26.0)	470 (16.1)	305 (10.4)	94 (3.2)	**2,922 (100.0)**
Vermont	—^¶^	108 (9.1)	305 (25.6)	322 (27.0)	247 (20.7)	153 (12.8)	—^¶^	**1,191 (99.7)**
Virginia	25 (0.2)	1,108 (7.1)	4,162 (26.7)	4,626 (29.7)	3,186 (20.4)	1,869 (12.0)	605 (3.9)	**15,581 (99.9)**
Washington	32 (0.2)	1,647 (9.6)	4,598 (26.7)	4,723 (27.4)	3,367 (19.5)	2,095 (12.2)	765 (4.4)	**17,227 (99.8)**
West Virginia	8 (0.7)	115 (9.7)	377 (31.9)	320 (27.0)	194 (16.4)	136 (11.5)	33 (2.8)	**1,183 (100.0)**
Wisconsin	17 (0.3)	690 (10.6)	1,917 (29.4)	1,806 (27.7)	1,202 (18.5)	637 (9.8)	242 (3.7)	**6,511 (100.0)**
Wyoming	—^¶^	—^¶^	6 (19.4)	10 (32.3)	8 (25.8)	6 (19.4)	—^¶^	**31 (100.0)**
**Total**	**1,410 (0.2)**	**53,049 (8.6)**	**171,043 (27.6)**	**181,052 (29.3)**	**121,279 (19.6)**	**67,835 (11.0)**	**23,121 (3.7)**	**618,789 (99.5)** ^§§^
**Abortion rate^¶¶^**	**0.4**	**6.0**	**19.0**	**18.6**	**13.0**	**7.4**	**2.7**	**NA**
Abortion ratio***	**873**	**348**	**275**	**194**	**132**	**145**	**224**	**NA**

**Abbreviation:** NA = not applicable.

* Data from 49 reporting areas; excludes three reporting areas (California, Maryland, and New Hampshire) that did not report, did not report by age, or did not meet reporting standards.

^†^ Percentages for the individual component categories might not add to 100% because of rounding.

^§^ Percentage is calculated as the number of abortions reported by known age divided by the sum of abortions reported by known and unknown age. Values ≥99.95% are rounded to 100.0%.

^¶^ Cells with a value in the range of 1–4 or cells that would allow for calculation of these small values have been suppressed.

** Includes residents only.

^††^ Reporting to the central health agency is not required. Data are requested from hospitals and licensed ambulatory care facilities only.

^§§^ Percentage based on a total of 622,137 abortions reported among the areas that met reporting standards for age; reporting standards for age were applied to abortions for residents of Illinois only.

^¶¶^ Number of abortions obtained by women in a given age group per 1,000 women in that same age group. Adolescents aged 13–14 years were used as the denominator for the group of adolescents aged <15 years, and women aged 40–44 years were used as the denominator for the group of women aged ≥40 years. For the total abortion rate only, abortions for women of unknown age were distributed according to the distribution of abortions among women of known age.

*** Number of abortions obtained by women in a given age group per 1,000 live births to women in that same age group. For the total abortion ratio only, abortions for women of unknown age were distributed according to the distribution of abortions among women of known age.

Among the 44 reporting areas that provided data each year by women’s age for 2010–2019, this pattern across age groups was stable, with the majority of abortions and the highest abortion rates occurring among women aged 20–29 years and the lowest percentages of abortions and abortion rates occurring among those in the youngest and oldest age groups (Table 4). From 2010 to 2019, abortion rates decreased among all age groups, although the decreases for adolescents (60% and 50% for adolescents aged <15 and 15–19 years) were greater than the decreases for all older age groups. From 2010 to 2014, the abortion rates decreased for all age groups, and from 2015 to 2019, the abortion rates decreased or did not change for all age groups except women aged 30–34 years and ≥40 years. From 2018 to 2019, abortion rates decreased or did not change for women aged ≤24 years; however, the abortion rate increased among those aged ≥25 years. During 2010–2019, abortion ratios decreased or did not change among all age groups, except for adolescents aged <15 years. The abortion ratio decreased for all age groups from 2010 to 2014; however, from 2015 to 2019, abortion ratios only decreased for women aged ≥35 years. From 2018 to 2019, abortion ratios increased for all age groups, except adolescents aged <15 years, for which it decreased.

**TABLE 4:** Percentage, rate,* and ratio^†^ of reported abortions, by known age group and year — selected reporting areas,^§^ United States, 2010–2019

Age group (yrs)	Year										% Change			
	2010	2011	2012	2013	2014	2015	2016	2017	2018	2019	2010 to 2014	2015 to 2019	2018 to 2019	2010 to 2019
**Reported abortions by known age (%)**														
<15	0.5	0.4	0.4	0.3	0.3	0.3	0.3	0.2	0.2	0.2	−40.0	−33.3	0.0	−60.0
15–19	14.6	13.5	12.2	11.4	10.4	9.8	9.4	9.1	8.8	8.7	−28.8	−11.2	−1.1	−40.4
20–24	32.9	32.9	32.8	32.7	32.1	31.1	30.0	29.3	28.5	27.8	−2.4	−10.6	−2.5	−15.5
25–29	24.5	24.9	25.4	25.9	26.8	27.6	28.5	29.0	29.4	29.3	9.4	6.2	−0.3	19.6
30–34	15.3	15.8	16.4	16.8	17.2	17.7	18.0	18.3	18.8	19.4	12.4	9.6	3.2	26.8
35–39	8.9	8.9	9.1	9.2	9.7	10.0	10.3	10.5	10.7	10.8	9.0	8.0	0.9	21.3
≥40	3.4	3.6	3.7	3.6	3.6	3.6	3.6	3.6	3.5	3.7	5.9	2.8	5.7	8.8
**Abortion rate**														
<15	1.0	0.9	0.8	0.6	0.5	0.5	0.4	0.4	0.4	0.4	−50.0	−20.0	0.0	−60.0
15–19	11.7	10.5	9.2	8.2	7.3	6.7	6.2	5.9	5.8	5.8	−37.6	−13.4	0.0	−50.4
20–24	26.8	25.0	23.3	21.9	20.9	19.9	19.0	18.4	18.2	18.1	−22.0	−9.0	−0.5	−32.5
25–29	20.2	19.5	18.9	18.2	18.1	17.9	17.7	17.3	17.6	17.8	−10.4	−0.6	1.1	−11.9
30–34	13.2	12.7	12.4	11.8	11.7	11.7	11.6	11.5	11.9	12.3	−11.4	5.1	3.4	−6.8
35–39	7.6	7.5	7.3	7.0	7.1	7.0	6.9	6.7	6.8	7.0	−6.6	0.0	2.9	−7.9
≥40	2.8	2.8	2.8	2.5	2.5	2.5	2.5	2.5	2.5	2.6	−10.7	4.0	4.0	−7.1
**Abortion ratio**														
<15	848	839	804	791	745	699	729	777	853	851	−12.1	21.7	−0.2	0.4
15–19	332	326	304	300	291	289	295	301	318	332	−12.3	14.9	4.4	0.0
20–24	291	284	272	262	256	250	249	249	256	260	−12.0	4.0	1.6	−10.7
25–29	184	178	174	169	167	167	169	171	178	183	−9.2	9.6	2.8	−0.5
30–34	138	132	128	122	116	115	113	114	119	124	−15.9	7.8	4.2	−10.1
35–39	171	165	158	148	145	140	136	134	135	137	−15.2	−2.1	1.5	−19.9
≥40	274	275	269	245	239	228	218	211	206	213	−12.8	−6.6	3.4	−22.3
Total (no.)^¶,^**	**672,271**	**640,719**	**611,540**	**579,406**	**565,691**	**553,596**	**542,922**	**528,308**	**533,557**	**539,634**	**NA**	**NA**	**NA**	**NA**

**Abbreviation:** NA = not applicable.

* Number of abortions obtained by women in a given age group per 1,000 women in that same age group. Adolescents aged 13–14 years were used as the denominator for the group of adolescents aged <15 years, and women aged 40–44 years were used as the denominator for the group of women aged ≥40 years. Abortions for women of unknown age were distributed according to the distribution of abortions among women of known age.

^†^ Number of abortions obtained by women in a given age group per 1,000 live births to women in that same age group. Abortions for women of unknown age were distributed according to the distribution of abortions among women of known age.

^§^ Data from 44 reporting areas; excludes eight reporting areas (California, District of Columbia, Florida, Maine, Maryland, New Hampshire, Vermont, and Wyoming) that did not report, did not report by age, or did not meet reporting standards for ≥1 year.

^¶^ By year, the total number of abortions represents 99.4%–99.7% of all abortions reported to CDC among the areas that met reporting standards for age during 2010–2019; reporting standards for age were applied to abortions for residents of Illinois only.

** The total number is different than previously reported because the totals by known age are presented and data for out-of-state residents were subsequently added for Wisconsin.

Among the 47 areas that reported age by individual year among adolescents for 2019, adolescents aged 18–19 years accounted for the majority (70.2%) of adolescent abortions and had the highest adolescent abortion rates (8.6 and 12.2 abortions per 1,000 adolescents aged 18 and 19 years, respectively) (Table 5). Adolescents aged <15 years accounted for the smallest percentage of adolescent abortions (2.6%) and had the lowest adolescent abortion rate (0.4 abortions per 1,000 adolescents aged 13–14 years). In 2019, the abortion ratio for adolescents was highest among adolescents aged <15 years (853 abortions per 1,000 live births) and was lowest among adolescents aged 17–19 years (344, 358, and 294 abortions per 1,000 live births among adolescents aged 17, 18, and 19 years).

**TABLE 5:** Number of reported abortions among adolescents, by known age and reporting area of occurrence — selected reporting areas,* United States, 2019

State/Area	Age group (yrs)						Total no.
	<15	15	16	17	18	19	
	No. (%)^†^	No. (%)	No. (%)	No. (%)	No. (%)	No. (%)	
Alabama	23 (4.2)	23 (4.2)	43 (7.8)	65 (11.8)	171 (31.1)	224 (40.8)	**549**
Alaska	—^§^	—^§^	—^§^	—^§^	—^§^	—^§^	**—** ^§^
Arizona	19 (1.6)	40 (3.4)	79 (6.7)	150 (12.7)	367 (31.0)	527 (44.6)	**1,182**
Arkansas	10 (3.3)	22 (7.2)	33 (10.9)	36 (11.8)	87 (28.6)	116 (38.2)	**304**
Colorado	27 (3.2)	39 (4.6)	62 (7.4)	130 (15.5)	259 (30.9)	322 (38.4)	**839**
Delaware	8 (3.2)	12 (4.8)	21 (8.5)	43 (17.3)	70 (28.2)	94 (37.9)	**248**
District of Columbia	10 (2.5)	19 (4.8)	35 (8.8)	74 (18.7)	99 (25.0)	159 (40.2)	**396**
Florida	118 (2.2)	199 (3.7)	402 (7.5)	689 (12.9)	1,585 (29.6)	2,356 (44.0)	**5,349**
Georgia	71 (2.4)	115 (4.0)	209 (7.2)	383 (13.2)	860 (29.6)	1,265 (43.6)	**2,903**
Hawaii	6 (3.1)	9 (4.6)	15 (7.7)	31 (15.8)	48 (24.5)	87 (44.4)	**196**
Idaho	—^§^	—^§^	—^§^	—^§^	—^§^	—^§^	**—** ^§^
Indiana	18 (2.3)	35 (4.5)	67 (8.5)	103 (13.1)	234 (29.8)	329 (41.9)	**786**
Iowa	12 (3.3)	24 (6.5)	36 (9.8)	49 (13.3)	108 (29.3)	140 (37.9)	**369**
Kansas	12 (1.9)	28 (4.3)	46 (7.1)	75 (11.6)	216 (33.5)	267 (41.5)	**644**
Kentucky	11 (3.6)	16 (5.2)	24 (7.9)	33 (10.8)	82 (26.9)	139 (45.6)	**305**
Louisiana	26 (3.5)	54 (7.2)	74 (9.8)	134 (17.8)	200 (26.6)	264 (35.1)	**752**
Maine	5 (2.5)	14 (6.9)	16 (7.9)	33 (16.3)	54 (26.6)	81 (39.9)	**203**
Massachusetts	28 (2.1)	43 (3.2)	100 (7.5)	169 (12.7)	383 (28.7)	611 (45.8)	**1,334**
Michigan	51 (2.1)	134 (5.6)	191 (8.0)	318 (13.4)	658 (27.7)	1,027 (43.2)	**2,379**
Minnesota	26 (3.1)	43 (5.1)	68 (8.0)	122 (14.4)	255 (30.0)	335 (39.5)	**849**
Mississippi	7 (2.3)	26 (8.6)	24 (7.9)	44 (14.6)	90 (29.8)	111 (36.8)	**302**
Missouri	5 (3.4)	15 (10.3)	12 (8.2)	27 (18.5)	37 (25.3)	50 (34.2)	**146**
Montana	5 (3.1)	10 (6.2)	17 (10.5)	26 (16.0)	42 (25.9)	62 (38.3)	**162**
Nebraska	7 (3.6)	10 (5.2)	15 (7.7)	34 (17.5)	37 (19.1)	91 (46.9)	**194**
Nevada	19 (2.7)	37 (5.2)	62 (8.7)	101 (14.2)	219 (30.8)	272 (38.3)	**710**
New Jersey^¶^	50 (2.5)	84 (4.2)	206 (10.3)	344 (17.1)	539 (26.8)	785 (39.1)	**2,008**
New Mexico	22 (4.2)	34 (6.4)	65 (12.3)	93 (17.6)	120 (22.7)	195 (36.9)	**529**
New York	186 (2.6)	316 (4.4)	657 (9.2)	1,218 (17.1)	2,026 (28.5)	2,702 (38.0)	**7,105**
New York City	109 (2.6)	192 (4.6)	406 (9.8)	735 (17.7)	1,170 (28.1)	1,549 (37.2)	**4,161**
New York State	77 (2.6)	124 (4.2)	251 (8.5)	483 (16.4)	856 (29.1)	1,153 (39.2)	**2,944**
North Carolina	60 (2.6)	112 (4.8)	186 (8.0)	282 (12.2)	689 (29.8)	983 (42.5)	**2,312**
North Dakota	0 (—)	6 (5.0)	13 (10.7)	16 (13.2)	33 (27.3)	53 (43.8)	**121**
Ohio	63 (3.5)	89 (4.9)	147 (8.2)	239 (13.3)	516 (28.7)	746 (41.4)	**1,800**
Oklahoma	89 (16.8)	19 (3.6)	29 (5.5)	67 (12.7)	145 (27.4)	180 (34.0)	**529**
Oregon	19 (2.2)	35 (4.1)	76 (8.9)	136 (15.9)	218 (25.5)	372 (43.5)	**856**
Pennsylvania	71 (2.8)	116 (4.6)	224 (8.8)	346 (13.6)	736 (28.9)	1,052 (41.3)	**2,545**
Rhode Island	—^§^	—^§^	—^§^	—^§^	—^§^	—^§^	**—** ^§^
South Carolina	10 (2.0)	16 (3.1)	42 (8.3)	115 (22.6)	138 (27.1)	188 (36.9)	**509**
South Dakota	—^§^	—^§^	—^§^	—^§^	—^§^	—^§^	**—** ^§^
Tennessee	17 (2.0)	38 (4.5)	76 (9.0)	108 (12.8)	238 (28.1)	370 (43.7)	**847**
Texas	106 (2.1)	213 (4.1)	426 (8.3)	677 (13.2)	1,449 (28.2)	2,276 (44.2)	**5,147**
Utah	6 (1.6)	13 (3.4)	23 (6.1)	49 (13.0)	119 (31.6)	167 (44.3)	**377**
Vermont	—^§^	—^§^	—^§^	—^§^	—^§^	—^§^	**—** ^§^
Virginia	25 (2.2)	57 (5.0)	82 (7.2)	138 (12.2)	325 (28.7)	506 (44.7)	**1,133**
Washington	32 (1.9)	74 (4.4)	145 (8.6)	277 (16.5)	477 (28.4)	674 (40.1)	**1,679**
West Virginia	8 (6.5)	5 (4.1)	7 (5.7)	19 (15.4)	35 (28.5)	49 (39.8)	**123**
Wisconsin**	17 (2.5)	27 (4.0)	73 (10.8)	77 (11.4)	195 (28.8)	289 (42.6)	**678**
Wyoming	—^§^	—^§^	—^§^	—^§^	—^§^	—^§^	**—** ^§^
**Total**	**1,315 (2.6)**	**2,248 (4.5)**	**4,175 (8.3)**	**7,148 (14.3)**	**14,378 (28.7)**	**20,791 (41.5)**	**50,055**
**Abortion rate** ^††^	**0.4**	**1.4**	**2.6**	**4.4**	**8.6**	**12.2**	**NA**
**Abortion ratio** ^§§^	**853**	**559**	**415**	**344**	**358**	**294**	**NA**

**Abbreviation:** NA = not applicable.

* Data from 47 reporting areas; excludes five reporting areas (California, Connecticut, Illinois, Maryland, and New Hampshire) that did not report, did not report age among adolescents by individual year, or did not meet reporting standards.

^†^ Percentages for the individual component categories might not add to 100% because of rounding.

^§^ Cells with a value in the range of 1–4 or cells that would allow for calculation of these small values have been suppressed.

^¶^ Reporting to the central health agency is not required. Data are requested from hospitals and licensed ambulatory care facilities only.

** Includes residents only.

^††^ Number of abortions obtained by women in a given age group per 1,000 women in that same age group. Adolescents aged 13–14 years were used as the denominator for the group of adolescents aged <15 years. For the total abortion rate only, abortions for women of unknown age were distributed according to the distribution of abortions among women of known age.

^§§^ Number of abortions obtained by women in a given age group per 1,000 live births to women in that same age group. For the total abortion ratio only, abortions for women of unknown age were distributed according to the distribution of abortions among women of known age.

Among the 30 areas that reported race by ethnicity data for 2019, non-Hispanic White women and non-Hispanic Black women accounted for the largest percentages of all abortions (33.4% and 38.4%, respectively), and Hispanic women and non-Hispanic women in the other race category accounted for smaller percentages (21.0% and 7.2%, respectively) (Table 6). Non-Hispanic White women had the lowest abortion rate (6.6 abortions per 1,000 women) and ratio (117 abortions per 1,000 live births), and non-Hispanic Black women had the highest abortion rate (23.8 abortions per 1,000 women) and ratio (386 abortions per 1,000 live births).

**TABLE 6:** Number of reported abortions, by known race/ethnicity and reporting area of occurrence — selected reporting areas,* United States, 2019

State/Area	Non-Hispanic			Hispanic	Total abortions reported by known race/ethnicity
	White	Black	Other		
	No. (%)^†^	No. (%)	No. (%)	No. (%)	No. (% of all reported abortions)^§^
Alabama	1,823 (30.4)	3,717 (61.9)	142 (2.4)	323 (5.4)	**6,005 (99.9)**
Alaska	584 (49.0)	85 (7.1)	470 (39.5)	52 (4.4)	**1,191 (93.8)**
Arizona	4,821 (38.4)	1,376 (10.9)	1,241 (9.9)	5,130 (40.8)	**12,568 (96.0)**
Arkansas	1,277 (44.0)	1,370 (47.2)	68 (2.3)	190 (6.5)	**2,905 (98.0)**
Connecticut	3,122 (37.4)	2,754 (33.0)	506 (6.1)	1,971 (23.6)	**8,353 (90.8)**
Delaware	863 (42.5)	850 (41.9)	72 (3.5)	244 (12.0)	**2,029 (99.4)**
District of Columbia	831 (18.8)	2,365 (53.4)	517 (11.7)	717 (16.2)	**4,430 (97.3)**
Florida	20,576 (30.9)	23,072 (34.6)	3,189 (4.8)	19,829 (29.7)	**66,666 (92.7)**
Georgia	7,102 (21.2)	21,709 (64.9)	1,724 (5.2)	2,924 (8.7)	**33,459 (90.7)**
Idaho	939 (67.5)	39 (2.8)	68 (4.9)	346 (24.9)	**1,392 (92.0)**
Indiana	3,919 (51.6)	2,404 (31.6)	583 (7.7)	691 (9.1)	**7,597 (99.5)**
Kansas	3,722 (54.0)	1,631 (23.7)	534 (7.8)	1,000 (14.5)	**6,887 (99.9)**
Kentucky	2,041 (55.7)	1,236 (33.7)	133 (3.6)	253 (6.9)	**3,663 (100.0)**
Michigan	10,578 (39.9)	13,687 (51.6)	1,201 (4.5)	1,041 (3.9)	**26,507 (97.0)**
Minnesota	4,310 (46.8)	2,569 (27.9)	1,392 (15.1)	937 (10.2)	**9,208 (92.6)**
Mississippi	619 (19.5)	2,352 (74.0)	114 (3.6)	95 (3.0)	**3,180 (99.6)**
Montana	1,293 (82.5)	—^¶^	142 (9.1)	—^¶^	**1,568 (100.0)**
Nevada	2,841 (37.0)	1,409 (18.4)	924 (12.0)	2,497 (32.6)	**7,671 (91.2)**
New Jersey**	5,133 (26.7)	7,453 (38.8)	3,206 (16.7)	3,427 (17.8)	**19,219 (86.7)**
New Mexico	930 (27.6)	160 (4.8)	380 (11.3)	1,897 (56.3)	**3,367 (85.4)**
North Carolina	8,096 (30.7)	12,968 (49.2)	1,990 (7.6)	3,298 (12.5)	**26,352 (92.6)**
South Carolina	2,481 (48.8)	1,986 (39.0)	232 (4.6)	390 (7.7)	**5,089 (99.8)**
South Dakota	259 (62.6)	46 (11.1)	69 (16.7)	40 (9.7)	**414 (100.0)**
Tennessee	3,688 (38.9)	4,842 (51.0)	363 (3.8)	595 (6.3)	**9,488 (97.6)**
Texas^††^	15,066 (26.3)	15,921 (27.8)	3,989 (7.0)	22,217 (38.8)	**57,193 (99.9)**
Utah	1,721 (60.4)	133 (4.7)	214 (7.5)	779 (27.4)	**2,847 (97.4)**
Vermont	1,045 (89.7)	47 (4.0)	41 (3.5)	32 (2.7)	**1,165 (97.5)**
Virginia	4,802 (33.6)	6,515 (45.5)	1,512 (10.6)	1,476 (10.3)	**14,305 (91.7)**
West Virginia	987 (83.4)	153 (12.9)	—^¶^	—^¶^	**1,183 (100.0)**
Wyoming	17 (60.7)	—^¶^	—^¶^	8 (28.6)	**28 (90.3)**
**Total**	**115,486 (33.4)**	**132,878 (38.4)**	**25,056 (7.2)**	**72,509 (21.0)**	**345,929 (94.5)^§§^**
**Abortion rate** ^¶¶^	**6.6**	**23.8**	**13.0**	**11.7**	**NA**
Abortion ratio***	**117**	**386**	**236**	**170**	**NA**

**Abbreviation:** NA = not applicable.

* Data from 30 reporting areas; excludes 22 reporting areas (California, Colorado, Hawaii, Illinois, Iowa, Louisiana, Maine, Maryland, Massachusetts, Missouri, Nebraska, New Hampshire, New York City, New York State, North Dakota, Ohio, Oklahoma, Oregon, Pennsylvania, Rhode Island, Washington, and Wisconsin) that did not report, did not report by race/ethnicity, or did not meet reporting standards.

^†^ Percentages for the individual component categories might not add to 100% because of rounding.

^§^ Percentage is calculated as the number of abortions reported by known race/ethnicity divided by the sum of abortions reported by known and unknown race/ethnicity. Values ≥99.95% are rounded to 100.0%.

^¶^ Cells with a value in the range of 1–4 or cells that would allow for calculation of these small values have been suppressed.

** Reporting to the central health agency is not required. Data are requested from hospitals and licensed ambulatory care facilities only.

^††^ Reporting form contains only one question for race/ethnicity; therefore, abortions reported for women of White, Black, and other races (Asian and Native American) are not explicitly identified as non-Hispanic.

^§§^ Percentage based on a total of 366,130 abortions reported among the areas that met reporting standards for race/ethnicity.

^¶¶^ Number of abortions obtained by women in a given racial/ethnic group per 1,000 women in that same racial/ethnic group. For the total abortion rate only, abortions for women of unknown race/ethnicity were distributed according to the distribution of abortions among women of known race/ethnicity.

*** Number of abortions obtained by women in a given racial/ethnic group per 1,000 live births to women in that same racial/ethnic group. For the total abortion ratio only, abortions for women of unknown race/ethnicity were distributed according to the distribution of abortions among women of known race/ethnicity.

Among the 42 areas that reported by marital status for 2019, 14.5% of women who obtained an abortion were married, and 85.5% were unmarried (Table 7). The abortion ratio was 46 abortions per 1,000 live births for married women and 394 abortions per 1,000 live births for unmarried women.

**TABLE 7:** Number of reported abortions, by known marital status and reporting area of occurrence — selected reporting areas,* United States, 2019

State/Area	Marital status		Total abortions reported by known marital status
	Married	Unmarried	
	No. (%)^†^	No. (%)	No. (% of all reported abortions)^§^
Alabama	663 (11.0)	5,342 (89.0)	**6,005 (99.9)**
Alaska	253 (21.1)	945 (78.9)	**1,198 (94.3)**
Arizona	1,878 (14.3)	11,219 (85.7)	**13,097 (100.0)**
Arkansas	376 (12.7)	2,575 (87.3)	**2,951 (99.6)**
Colorado	1,559 (19.1)	6,596 (80.9)	**8,155 (90.6)**
Connecticut	939 (11.5)	7,232 (88.5)	**8,171 (88.8)**
Delaware	224 (11.0)	1,818 (89.0)	**2,042 (100.0)**
Florida	10,136 (16.1)	52,629 (83.9)	**62,765 (87.3)**
Georgia	4,410 (12.8)	30,033 (87.2)	**34,443 (93.3)**
Idaho	302 (21.6)	1,094 (78.4)	**1,396 (92.3)**
Illinois^¶^	3,575 (9.5)	33,868 (90.5)	**37,443 (96.6)**
Indiana	1,094 (14.3)	6,543 (85.7)	**7,637 (100.0)**
Iowa	633 (17.8)	2,925 (82.2)	**3,558 (99.8)**
Kansas	1,063 (15.5)	5,811 (84.5)	**6,874 (99.7)**
Kentucky	525 (14.3)	3,139 (85.7)	**3,664 (100.0)**
Louisiana	828 (10.5)	7,063 (89.5)	**7,891 (96.9)**
Maine	308 (16.0)	1,612 (84.0)	**1,920 (95.0)**
Michigan	2,660 (10.3)	23,095 (89.7)	**25,755 (94.2)**
Minnesota	1,535 (16.2)	7,959 (83.8)	**9,494 (95.5)**
Mississippi	290 (9.1)	2,893 (90.9)	**3,183 (99.7)**
Missouri	224 (16.0)	1,179 (84.0)	**1,403 (95.4)**
Montana	286 (18.2)	1,282 (81.8)	**1,568 (100.0)**
Nebraska	306 (15.0)	1,732 (85.0)	**2,038 (98.5)**
New Jersey**	2,515 (11.5)	19,372 (88.5)	**21,887 (98.7)**
New Mexico	563 (15.3)	3,128 (84.7)	**3,691 (93.6)**
New York City	8,269 (18.5)	36,518 (81.5)	**44,787 (90.0)**
North Carolina	3,622 (14.4)	21,547 (85.6)	**25,169 (88.5)**
North Dakota	162 (14.5)	958 (85.5)	**1,120 (99.9)**
Ohio	2,603 (14.0)	16,027 (86.0)	**18,630 (92.7)**
Oklahoma	941 (18.9)	4,043 (81.1)	**4,984 (99.8)**
Oregon	1,573 (20.3)	6,193 (79.7)	**7,766 (89.4)**
Pennsylvania	3,766 (12.2)	27,221 (87.8)	**30,987 (99.9)**
Rhode Island	263 (12.9)	1,782 (87.1)	**2,045 (97.4)**
South Carolina	726 (14.4)	4,331 (85.6)	**5,057 (99.1)**
South Dakota	85 (20.5)	329 (79.5)	**414 (100.0)**
Tennessee	1,295 (14.0)	7,985 (86.0)	**9,280 (95.5)**
Texas	9,784 (17.1)	47,363 (82.9)	**57,147 (99.8)**
Utah	724 (24.9)	2,182 (75.1)	**2,906 (99.5)**
Vermont	216 (20.3)	847 (79.7)	**1,063 (89.0)**
Virginia^††^	2,349 (15.1)	13,252 (84.9)	**15,601 (100.0)**
West Virginia	203 (17.2)	979 (82.8)	**1,182 (99.9)**
Wisconsin	852 (13.2)	5,615 (86.8)	**6,467 (99.3)**
**Total**	**74,578 (14.5)**	**438,256 (85.5)**	**512,834 (94.5)^§§^**
**Abortion ratio^¶¶^**	**46**	**394**	**NA**

**Abbreviation:** NA = not applicable.

* Data from 42 reporting areas; excludes 10 reporting areas (California, District of Columbia, Hawaii, Maryland, Massachusetts, Nevada, New Hampshire, New York State, Washington, and Wyoming) that did not report, did not report by marital status, or did not meet reporting standards.

^†^ Percentages for the individual component categories might not add to 100% because of rounding.

^§^ Percentage is calculated as the number of abortions reported by known marital status divided by the sum of abortions reported by known and unknown marital status. Values ≥99.95% are rounded to 100.0%.

^¶^ Includes residents only.

** Reporting to the central health agency is not required. Data are requested from hospitals and licensed ambulatory care facilities only.

^††^ Recorded as patient married or not married to father.

^§§^ Percentage based on a total of 542,479 abortions reported among the areas that met reporting standards for marital status; reporting standards for marital status were applied to abortions for residents of Illinois only.

^¶¶^ Number of abortions obtained by marital status per 1,000 live births to women of the same marital status. For the total abortion ratio only, abortions for women of unknown marital status were distributed according to the distribution of abortions among women of known marital status.

### Previous Live Births and Previous Induced Abortions

Among the 45 areas that reported the number of previous live births for 2019, 40.2%, 24.5%, 20.0%, 9.2%, and 6.0% of women had zero, one, two, three, or four or more previous live births (Table 8). Among the 44 areas that reported the number of previous induced abortions for 2019, the majority of women (58.2%) had previously had no abortions, 23.8% had previously had one abortion, 10.5% had previously had two abortions, and 7.5% had previously had three or more abortions (Table 9).

**TABLE 8:** Number of reported abortions, by known number of previous live births and reporting area of occurrence — selected reporting areas,* United States, 2019

State/Area	No. of previous live births					Total abortions reported by known number of previous live births
	0	1	2	3	≥4	
	No. (%)^†^	No. (%)	No. (%)	No. (%)	No. (%)	No. (% of all reported abortions)^§^
Alabama	2,030 (33.8)	1,660 (27.6)	1,369 (22.8)	602 (10.0)	348 (5.8)	**6,009 (100.0)**
Alaska	575 (45.3)	233 (18.4)	258 (20.3)	103 (8.1)	100 (7.9)	**1,269 (99.9)**
Arizona	5,707 (44.0)	2,784 (21.5)	2,426 (18.7)	1,195 (9.2)	846 (6.5)	**12,958 (98.9)**
Arkansas	1,042 (35.2)	771 (26.0)	650 (21.9)	295 (10.0)	205 (6.9)	**2,963 (100.0)**
Colorado	5,167 (57.7)	1,644 (18.3)	1,268 (14.1)	573 (6.4)	310 (3.5)	**8,962 (99.6)**
Connecticut	3,731 (40.6)	2,319 (25.2)	1,833 (20.0)	839 (9.1)	465 (5.1)	**9,187 (99.8)**
Delaware	849 (42.1)	501 (24.8)	390 (19.3)	159 (7.9)	118 (5.9)	**2,017 (98.8)**
Florida	27,510 (38.3)	18,129 (25.2)	14,878 (20.7)	6,660 (9.3)	4,737 (6.6)	**71,914 (100.0)**
Georgia	14,313 (38.8)	9,116 (24.7)	7,405 (20.1)	3,646 (9.9)	2,425 (6.6)	**36,905 (100.0)**
Hawaii	1,096 (54.8)	338 (16.9)	313 (15.7)	160 (8.0)	92 (4.6)	**1,999 (99.8)**
Idaho	694 (46.3)	316 (21.1)	277 (18.5)	130 (8.7)	83 (5.5)	**1,500 (99.1)**
Indiana	3,045 (39.9)	1,831 (24.0)	1,558 (20.4)	780 (10.2)	423 (5.5)	**7,637 (100.0)**
Iowa	1,430 (40.4)	781 (22.1)	705 (19.9)	356 (10.1)	268 (7.6)	**3,540 (99.3)**
Kansas	2,795 (40.5)	1,686 (24.5)	1,327 (19.2)	681 (9.9)	405 (5.9)	**6,894 (100.0)**
Kentucky	1,300 (35.5)	934 (25.5)	871 (23.8)	338 (9.2)	221 (6.0)	**3,664 (100.0)**
Louisiana	2,627 (32.3)	2,231 (27.5)	1,807 (22.2)	894 (11.0)	564 (6.9)	**8,123 (99.7)**
Maine	1,002 (49.7)	431 (21.4)	362 (18.0)	148 (7.3)	73 (3.6)	**2,016 (99.8)**
Massachusetts	7,765 (46.2)	3,910 (23.3)	3,147 (18.7)	1,324 (7.9)	645 (3.8)	**16,791 (90.3)**
Michigan^¶^	9,472 (34.7)	7,075 (25.9)	6,209 (22.7)	2,827 (10.3)	1,748 (6.4)	**27,331 (100.0)**
Minnesota	3,954 (40.1)	2,265 (23.0)	1,944 (19.7)	964 (9.8)	730 (7.4)	**9,857 (99.2)**
Mississippi	996 (31.2)	924 (28.9)	714 (22.4)	342 (10.7)	218 (6.8)	**3,194 (100.0)**
Missouri	561 (38.1)	353 (24.0)	301 (20.5)	146 (9.9)	110 (7.5)	**1,471 (100.0)**
Montana	746 (47.6)	357 (22.8)	260 (16.6)	129 (8.2)	76 (4.8)	**1,568 (100.0)**
Nebraska	819 (39.6)	445 (21.5)	429 (20.8)	216 (10.4)	158 (7.6)	**2,067 (100.0)**
Nevada	3,606 (42.9)	1,954 (23.2)	1,496 (17.8)	790 (9.4)	567 (6.7)	**8,413 (100.0)**
New Jersey**	8,148 (36.9)	6,140 (27.8)	4,303 (19.5)	2,105 (9.5)	1,411 (6.4)	**22,107 (99.7)**
New Mexico	1,502 (42.6)	801 (22.7)	621 (17.6)	357 (10.1)	248 (7.0)	**3,529 (89.5)**
New York City	20,793 (45.8)	11,596 (25.5)	8,380 (18.4)	2,987 (6.6)	1,680 (3.7)	**45,436 (91.3)**
North Carolina	9,498 (37.4)	6,067 (23.9)	5,016 (19.8)	2,660 (10.5)	2,154 (8.5)	**25,395 (89.3)**
North Dakota	452 (40.3)	243 (21.7)	223 (19.9)	118 (10.5)	85 (7.6)	**1,121 (100.0)**
Ohio^††^	7,074 (36.1)	4,963 (25.3)	4,176 (21.3)	2,049 (10.4)	1,360 (6.9)	**19,622 (97.6)**
Oklahoma	1,890 (37.9)	1,208 (24.2)	1,055 (21.1)	507 (10.2)	332 (6.7)	**4,992 (99.9)**
Oregon	4,366 (50.6)	1,759 (20.4)	1,501 (17.4)	590 (6.8)	407 (4.7)	**8,623 (99.3)**
Pennsylvania	11,760 (37.9)	7,926 (25.6)	6,367 (20.5)	3,046 (9.8)	1,919 (6.2)	**31,018 (100.0)**
Rhode Island	963 (45.9)	506 (24.1)	393 (18.7)	144 (6.9)	92 (4.4)	**2,098 (100.0)**
South Carolina	2,129 (41.7)	1,294 (25.4)	1,027 (20.1)	409 (8.0)	242 (4.7)	**5,101 (100.0)**
South Dakota	165 (39.9)	72 (17.4)	94 (22.7)	—^§§^	—^§§^	**414 (100.0)**
Tennessee	3,484 (36.3)	2,498 (26.0)	1,974 (20.6)	940 (9.8)	695 (7.2)	**9,591 (98.7)**
Texas	22,862 (39.9)	13,628 (23.8)	11,985 (20.9)	5,483 (9.6)	3,317 (5.8)	**57,275 (100.0)**
Utah	1,496 (51.2)	555 (19.0)	470 (16.1)	230 (7.9)	171 (5.9)	**2,922 (100.0)**
Vermont	597 (50.0)	235 (19.7)	231 (19.3)	88 (7.4)	43 (3.6)	**1,194 (99.9)**
Virginia	5,531 (35.5)	4,095 (26.2)	3,326 (21.3)	1,646 (10.6)	1,003 (6.4)	**15,601 (100.0)**
Washington	8,035 (46.7)	3,845 (22.3)	3,163 (18.4)	1,370 (8.0)	805 (4.7)	**17,218 (99.7)**
West Virginia	421 (35.6)	347 (29.4)	255 (21.6)	97 (8.2)	62 (5.2)	**1,182 (99.9)**
Wyoming	17 (54.8)	5 (16.1)	7 (22.6)	—^§§^	—^§§^	**31 (100.0)**
**Total**	**214,015 (40.2)**	**130,771 (24.5)**	**106,764 (20.0)**	**49,178 (9.2)**	**31,991 (6.0)**	**532,719 (98.0)^¶¶^**

* Data from 45 reporting areas; excludes seven reporting areas (California, District of Columbia, Illinois, Maryland, New Hampshire, New York State, and Wisconsin) that did not report, did not report by number of previous live births, or did not meet reporting standards.

^†^ Percentages for the individual component categories might not add to 100% because of rounding.

^§^ Percentage is calculated as the number of abortions reported by known number of previous live births divided by the sum of abortions reported by known and unknown number of previous live births. Values ≥99.95% are rounded to 100.0%.

^¶^ Recorded as the number of previous pregnancies carried to term.

** Reporting to the central health agency is not required. Data are requested from hospitals and licensed ambulatory care facilities only.

^††^ Recorded as the number of living children.

^§§^ Cells with a value in the range of 1–4 or cells that would allow for calculation of these small values have been suppressed.

^¶¶^ Percentage based on a total of 543,515 abortions reported among the areas that met reporting standards for the number of previous live births.

**TABLE 9:** Number of reported abortions, by known number of previous induced abortions and reporting area of occurrence — selected reporting areas,* United States, 2019

State/Area	No. of previous induced abortions				Total abortions reported by known number of previous induced abortions
	0	1	2	≥3	
	No. (%)^†^	No. (%)	No. (%)	No. (%)	No. (% of all reported abortions)^§^
Alabama	4,008 (66.7)	1,332 (22.2)	443 (7.4)	226 (3.8)	**6,009 (100.0)**
Alaska	819 (64.5)	271 (21.4)	109 (8.6)	70 (5.5)	**1,269 (99.9)**
Arizona	8,472 (65.6)	3,047 (23.6)	955 (7.4)	445 (3.4)	**12,919 (98.6)**
Arkansas	1,915 (64.6)	587 (19.8)	233 (7.9)	228 (7.7)	**2,963 (100.0)**
Colorado	6,287 (69.9)	1,852 (20.6)	601 (6.7)	251 (2.8)	**8,991 (99.9)**
Connecticut	4,883 (53.2)	2,163 (23.5)	1,123 (12.2)	1,018 (11.1)	**9,187 (99.8)**
Delaware	1,244 (61.5)	472 (23.3)	197 (9.7)	109 (5.4)	**2,022 (99.0)**
Florida	41,693 (58.0)	17,171 (23.9)	7,405 (10.3)	5,645 (7.8)	**71,914 (100.0)**
Georgia	22,534 (61.1)	8,778 (23.8)	3,605 (9.8)	1,990 (5.4)	**36,907 (100.0)**
Hawaii	1,244 (62.2)	452 (22.6)	178 (8.9)	125 (6.3)	**1,999 (99.8)**
Idaho	1,228 (81.8)	209 (13.9)	44 (2.9)	21 (1.4)	**1,502 (99.3)**
Indiana	5,160 (67.6)	1,712 (22.4)	539 (7.1)	226 (3.0)	**7,637 (100.0)**
Iowa	2,675 (75.6)	565 (16.0)	186 (5.3)	114 (3.2)	**3,540 (99.3)**
Kansas	4,647 (67.4)	1,467 (21.3)	499 (7.2)	281 (4.1)	**6,894 (100.0)**
Kentucky	2,324 (63.4)	866 (23.6)	293 (8.0)	181 (4.9)	**3,664 (100.0)**
Louisiana	5,098 (62.7)	2,006 (24.7)	732 (9.0)	291 (3.6)	**8,127 (99.8)**
Maine	1,314 (65.1)	455 (22.6)	153 (7.6)	95 (4.7)	**2,017 (99.8)**
Massachusetts	9,507 (52.6)	4,868 (26.9)	2,240 (12.4)	1,466 (8.1)	**18,081 (97.2)**
Michigan	14,134 (51.7)	6,797 (24.9)	3,684 (13.5)	2,712 (9.9)	**27,327 (100.0)**
Minnesota	6,030 (61.1)	2,197 (22.3)	937 (9.5)	708 (7.2)	**9,872 (99.3)**
Mississippi	2,159 (67.6)	685 (21.4)	249 (7.8)	101 (3.2)	**3,194 (100.0)**
Missouri	882 (60.0)	367 (24.9)	141 (9.6)	81 (5.5)	**1,471 (100.0)**
Montana	643 (41.0)	622 (39.7)	199 (12.7)	104 (6.6)	**1,568 (100.0)**
Nebraska	1,429 (69.1)	430 (20.8)	142 (6.9)	67 (3.2)	**2,068 (100.0)**
Nevada	5,281 (62.8)	1,874 (22.3)	744 (8.8)	512 (6.1)	**8,411 (100.0)**
New Jersey^¶^	13,486 (60.9)	4,195 (18.9)	2,251 (10.2)	2,223 (10.0)	**22,155 (99.9)**
New York City	16,911 (37.9)	11,024 (24.7)	8,392 (18.8)	8,244 (18.5)	**44,571 (89.5)**
North Carolina	15,400 (61.4)	5,874 (23.4)	2,474 (9.9)	1,329 (5.3)	**25,077 (88.1)**
North Dakota	778 (70.7)	200 (18.2)	92 (8.4)	31 (2.8)	**1,101 (98.2)**
Ohio	11,689 (59.7)	4,727 (24.2)	1,915 (9.8)	1,242 (6.3)	**19,573 (97.4)**
Oklahoma	3,558 (71.3)	975 (19.5)	304 (6.1)	155 (3.1)	**4,992 (99.9)**
Oregon	5,182 (60.1)	2,064 (23.9)	809 (9.4)	574 (6.7)	**8,629 (99.3)**
Pennsylvania	16,327 (52.6)	7,699 (24.8)	3,829 (12.3)	3,163 (10.2)	**31,018 (100.0)**
Rhode Island	1,286 (61.4)	480 (22.9)	218 (10.4)	110 (5.3)	**2,094 (99.8)**
South Carolina	3,320 (65.1)	1,160 (22.7)	414 (8.1)	207 (4.1)	**5,101 (100.0)**
South Dakota	285 (68.8)	88 (21.3)	—**	—**	**414 (100.0)**
Tennessee	6,230 (64.7)	2,326 (24.2)	724 (7.5)	348 (3.6)	**9,628 (99.1)**
Texas	35,902 (62.7)	14,039 (24.5)	4,874 (8.5)	2,460 (4.3)	**57,275 (100.0)**
Utah	2,251 (77.0)	506 (17.3)	114 (3.9)	51 (1.7)	**2,922 (100.0)**
Vermont	746 (62.5)	266 (22.3)	111 (9.3)	70 (5.9)	**1,193 (99.8)**
Virginia	8,520 (54.6)	4,322 (27.7)	1,670 (10.7)	1,089 (7.0)	**15,601 (100.0)**
Washington	9,911 (57.6)	4,219 (24.5)	1,713 (10.0)	1,363 (7.9)	**17,206 (99.7)**
West Virginia	728 (61.5)	298 (25.2)	106 (9.0)	51 (4.3)	**1,183 (100.0)**
Wyoming	20 (64.5)	9 (29.0)	—**	—**	**31 (100.0)**
**Total**	**308,140 (58.2)**	**125,716 (23.8)**	**55,669 (10.5)**	**39,792 (7.5)**	**529,317 (98.1)^††^**

* Data from 44 reporting areas; excludes eight reporting areas (California, District of Columbia, Illinois, Maryland, New Hampshire, New Mexico, New York State, and Wisconsin) that did not report, did not report by number of previous induced abortions, or did not meet reporting standards.

^†^ Percentages for the individual component categories might not add to 100% because of rounding.

^§^ Percentage is calculated as the number of abortions reported by known number of previous induced abortions divided by the sum of abortions reported by known and unknown number of previous induced abortions. Values ≥99.95% are rounded to 100.0%.

^¶^ Reporting to the central health agency is not required. Data are requested from hospitals and licensed ambulatory care facilities only.

** Cells with a value in the range of 1–4 or cells that would allow for calculation of these small values have been suppressed.

**^††^** Percentage based on a total of 539,573 abortions reported among the areas that met reporting standards for the number of previous induced abortions.

### Weeks of Gestation and Method Type

Among the 43 areas that reported gestational age at the time of abortion for 2019, 79.3% of abortions were performed at ≤9 weeks’ gestation, and nearly all (92.7%) were performed at ≤13 weeks’ gestation (Table 10). Fewer abortions were performed at 14–20 weeks’ gestation (6.2%) or at ≥21 weeks’ gestation (<1.0%). Among the 34 reporting areas that provided data every year on gestational age for 2010–2019, the percentage of abortions performed at ≤13 weeks’ gestation changed negligibly, from 91.9% to 92.0% (Table 11). However, within this gestational age range, a shift occurred toward earlier gestational ages, with the percentage of abortions performed at ≤6 weeks’ gestation increasing 8% and the percentage of abortions performed at 7–9 weeks’ and 10–13 weeks’ gestation decreasing 0.5% and 14%, respectively.

**TABLE 10:** Number of reported abortions, by known weeks of gestation* and reporting area of occurrence — selected reporting areas,^†^ United States, 2019

State/Area	Weeks of gestation							Total abortions reported by known gestational age
	≤6	7–9	10–13	14–15	16–17	18–20	≥21	
	No. (%)^§^	No. (%)	No. (%)	No. (%)	No. (%)	No. (%)	No. (%)	No. (% of all reported abortions)^¶^
Alabama**	1,280 (21.3)	2,807 (46.8)	1,257 (20.9)	317 (5.3)	163 (2.7)	133 (2.2)	46 (0.8)	**6,003 (99.9)**
Alaska	292 (23.0)	639 (50.4)	271 (21.4)	64 (5.0)	—^††^	—^††^	0 (—)	**1,269 (99.9)**
Arizona	3,863 (29.5)	5,985 (45.7)	2,079 (15.9)	496 (3.8)	261 (2.0)	245 (1.9)	168 (1.3)	**13,097 (100.0)**
Arkansas**	479 (16.2)	1,265 (42.7)	862 (29.1)	150 (5.1)	82 (2.8)	97 (3.3)	28 (0.9)	**2,963 (100.0)**
Colorado	3,639 (40.4)	3,666 (40.7)	1,062 (11.8)	198 (2.2)	150 (1.7)	110 (1.2)	173 (1.9)	**8,998 (100.0)**
Connecticut	4,046 (45.7)	3,155 (35.7)	947 (10.7)	263 (3.0)	175 (2.0)	164 (1.9)	95 (1.1)	**8,845 (96.1)**
Delaware	536 (26.3)	1,071 (52.5)	328 (16.1)	73 (3.6)	16 (0.8)	6 (0.3)	9 (0.4)	**2,039 (99.9)**
Florida	52,850 (73.5)	11,641 (16.2)	4,843 (6.7)	973 (1.4)	691 (1.0)	699 (1.0)	217 (0.3)	**71,914 (100.0)**
Georgia	16,086 (43.6)	13,864 (37.6)	4,396 (11.9)	927 (2.5)	653 (1.8)	752 (2.0)	229 (0.6)	**36,907 (100.0)**
Hawaii	678 (33.9)	861 (43.0)	268 (13.4)	80 (4.0)	43 (2.1)	49 (2.4)	22 (1.1)	**2,001 (99.9)**
Idaho	493 (32.9)	707 (47.1)	257 (17.1)	37 (2.5)	—^††^	—^††^	—^††^	**1,500 (99.1)**
Indiana	1,924 (25.2)	4,055 (53.1)	1,618 (21.2)	9 (0.1)	8 (0.1)	17 (0.2)	6 (0.1)	**7,637 (100.0)**
Iowa	1,652 (46.3)	1,305 (36.6)	412 (11.6)	68 (1.9)	58 (1.6)	54 (1.5)	17 (0.5)	**3,566 (100.0)**
Kansas	2,761 (40.0)	2,722 (39.5)	921 (13.4)	195 (2.8)	121 (1.8)	137 (2.0)	37 (0.5)	**6,894 (100.0)**
Kentucky	1,302 (35.5)	1,493 (40.7)	550 (15.0)	116 (3.2)	65 (1.8)	109 (3.0)	29 (0.8)	**3,664 (100.0)**
Louisiana	2,815 (34.6)	3,567 (43.8)	1,274 (15.7)	273 (3.4)	173 (2.1)	38 (0.5)	0 (—)	**8,140 (100.0)**
Maine	595 (29.5)	996 (49.3)	317 (15.7)	48 (2.4)	33 (1.6)	31 (1.5)	0 (—)	**2,020 (100.0)**
Michigan	9,016 (33.0)	11,496 (42.1)	4,055 (14.9)	1,110 (4.1)	667 (2.4)	584 (2.1)	353 (1.3)	**27,281 (99.8)**
Minnesota	3,597 (36.7)	3,845 (39.2)	1,381 (14.1)	379 (3.9)	194 (2.0)	216 (2.2)	187 (1.9)	**9,799 (98.6)**
Mississippi	1,117 (35.0)	1,421 (44.5)	468 (14.7)	171 (5.4)	16 (0.5)	—^††^	—^††^	**3,194 (100.0)**
Missouri	86 (5.8)	496 (33.7)	505 (34.3)	130 (8.8)	87 (5.9)	112 (7.6)	55 (3.7)	**1,471 (100.0)**
Montana	599 (38.2)	628 (40.1)	211 (13.5)	51 (3.3)	34 (2.2)	34 (2.2)	11 (0.7)	**1,568 (100.0)**
Nebraska	976 (47.2)	683 (33.0)	284 (13.7)	62 (3.0)	46 (2.2)	16 (0.8)	0 (—)	**2,067 (100.0)**
Nevada	3,214 (38.6)	3,510 (42.1)	1,078 (12.9)	250 (3.0)	142 (1.7)	89 (1.1)	52 (0.6)	**8,335 (99.1)**
New Jersey^§§^	8,513 (39.3)	7,499 (34.6)	2,923 (13.5)	961 (4.4)	638 (2.9)	613 (2.8)	514 (2.4)	**21,661 (97.7)**
New Mexico	1,487 (42.7)	957 (27.5)	381 (10.9)	80 (2.3)	73 (2.1)	101 (2.9)	406 (11.6)	**3,485 (88.4)**
New York City	22,364 (44.9)	17,579 (35.3)	5,579 (11.2)	1,335 (2.7)	897 (1.8)	934 (1.9)	1,096 (2.2)	**49,784 (100.0)**
North Carolina	9,598 (33.9)	12,098 (42.8)	4,432 (15.7)	982 (3.5)	672 (2.4)	484 (1.7)	15 (0.1)	**28,281 (99.4)**
North Dakota	435 (38.8)	447 (39.9)	180 (16.1)	42 (3.7)	17 (1.5)	0 (—)	0 (—)	**1,121 (100.0)**
Ohio	5,523 (27.5)	9,070 (45.1)	3,558 (17.7)	848 (4.2)	531 (2.6)	477 (2.4)	95 (0.5)	**20,102 (100.0)**
Oklahoma	2,177 (43.6)	1,835 (36.8)	710 (14.2)	125 (2.5)	64 (1.3)	64 (1.3)	16 (0.3)	**4,991 (99.9)**
Oregon	4,064 (47.2)	2,924 (33.9)	949 (11.0)	241 (2.8)	129 (1.5)	149 (1.7)	160 (1.9)	**8,616 (99.2)**
Rhode Island	929 (44.4)	705 (33.7)	270 (12.9)	90 (4.3)	52 (2.5)	32 (1.5)	13 (0.6)	**2,091 (99.6)**
South Carolina**	1,063 (20.8)	1,970 (38.6)	1,740 (34.1)	298 (5.8)	8 (0.2)	13 (0.3)	9 (0.2)	**5,101 (100.0)**
South Dakota	64 (15.6)	224 (54.8)	—^††^	—^††^	0 (—)	—^††^	7 (1.7)	**409 (98.8)**
Tennessee	1,836 (18.9)	4,939 (50.9)	2,188 (22.5)	436 (4.5)	176 (1.8)	119 (1.2)	9 (0.1)	**9,703 (99.8)**
Texas**	22,356 (39.0)	22,721 (39.7)	8,232 (14.4)	1,870 (3.3)	957 (1.7)	838 (1.5)	301 (0.5)	**57,275 (100.0)**
Utah	1,018 (34.8)	1,185 (40.6)	478 (16.4)	92 (3.1)	51 (1.7)	67 (2.3)	31 (1.1)	**2,922 (100.0)**
Vermont	550 (46.0)	423 (35.4)	129 (10.8)	32 (2.7)	21 (1.8)	22 (1.8)	18 (1.5)	**1,195 (100.0)**
Virginia	7,917 (50.8)	5,215 (33.5)	1,938 (12.4)	121 (0.8)	131 (0.8)	170 (1.1)	90 (0.6)	**15,582 (99.9)**
Washington	7,046 (41.0)	6,768 (39.4)	2,061 (12.0)	420 (2.4)	265 (1.5)	273 (1.6)	363 (2.1)	**17,196 (99.6)**
West Virginia	325 (27.5)	536 (45.3)	235 (19.9)	58 (4.9)	18 (1.5)	—^††^	—^††^	**1,183 (100.0)**
Wyoming	18 (58.1)	12 (38.7)	—^††^	—^††^	0 (—)	0 (—)	0 (—)	**31 (100.0)**
**Total**	**211,179 (42.9)**	**178,985 (36.4)**	**65,739 (13.4)**	**14,471 (2.9)**	**8,581 (1.7)**	**8,064 (1.6)**	**4,882 (1.0)**	**491,901 (99.6)^¶¶^**

* Gestational age based on clinician’s estimate (Alaska, Arizona, Colorado, Connecticut, Delaware, Florida, Georgia, Hawaii, Idaho, Indiana, Iowa, Kansas, Kentucky, Louisiana, Maine, Michigan, Minnesota, Mississippi, Missouri, Montana, Nebraska, Nevada, New Jersey, New Mexico, New York City, North Carolina, North Dakota, Ohio, Oregon, Rhode Island, South Dakota, Tennessee, Vermont, Washington, West Virginia, and Wyoming); gestational age calculated from the last normal menstrual period (Oklahoma and Utah); clinician’s estimate of gestation based on estimated date of conception (Virginia); probable postfertilization age (Alabama, Arkansas, South Carolina, and Texas).

^†^ Data from 43 reporting areas; excludes nine reporting areas (California, District of Columbia, Illinois, Maryland, Massachusetts, New Hampshire, New York State, Pennsylvania, and Wisconsin) that did not report, did not report by gestational age, or did not meet reporting standards.

^§^ Percentages for the individual component categories might not add to 100% because of rounding.

^¶^ Percentage is calculated as the number of abortions reported by known gestational age divided by the sum of abortions reported by known and unknown gestational age. Values ≥99.95% are rounded to 100.0%.

** Two weeks were added to the probable postfertilization age to provide a corresponding measure to gestational age based on the clinician’s estimate.

^††^ Cells with a value in the range of 1–4 or cells that would allow for calculation of these small values have been suppressed.

^§§^ Reporting to the central health agency is not required. Data are requested from hospitals and licensed ambulatory care facilities only.

^¶¶^ Percentage based on a total of 493,904 abortions reported among the areas that met reporting standards for gestational age.

**TABLE 11:** Percentage of reported abortions, by known weeks of gestation and year — selected reporting areas,* United States, 2010–2019

Weeks of gestation	Year										% Change			
	2010	2011	2012	2013	2014	2015	2016	2017	2018	2019	2010 to 2014	2015 to 2019	2018 to 2019	2010 to 2019
**≤13 weeks’ gestation (%)^†^**	**91.9**	**91.5**	**91.4**	**91.6**	**91.0**	**91.0**	**91.0**	**91.1**	**91.5**	**92.0**	**−1.0**	**1.1**	**0.5**	**0.1**
≤6	34.7	34.3	35.1	34.7	33.8	34.3	34.2	35.1	36.2	37.5	−2.6	9.3	3.6	8.1
7–9	40.1	40.1	39.4	39.9	40.0	40.0	40.3	40.4	40.0	39.9	−0.2	−0.3	−0.3	−0.5
10–13	17.0	17.1	16.9	17.0	17.2	16.7	16.4	15.7	15.2	14.6	1.2	−12.6	−3.9	−14.1
**>13 weeks’ gestation (%)^†^**	**8.1**	**8.5**	**8.6**	**8.4**	**9.0**	**9.0**	**9.0**	**8.9**	**8.5**	**8.0**	**11.1**	**−11.1**	**−5.9**	**−1.2**
14–15	3.3	3.4	3.5	3.4	3.5	3.5	3.6	3.4	3.4	3.2	6.1	−8.6	−5.9	−3.0
16–17	1.8	1.9	1.9	1.9	2.2	2.1	2.1	2.2	2.1	1.9	22.2	−9.5	−9.5	5.6
18–20	1.8	1.9	1.9	1.8	1.9	2.0	2.0	2.0	1.9	1.8	5.6	−10.0	−5.3	0.0
≥21	1.2	1.4	1.3	1.3	1.3	1.3	1.3	1.3	1.2	1.1	8.3	−15.4	−8.3	−8.3
**Total (no.)^§^**	**508,841**	**481,667**	**457,201**	**435,881**	**426,636**	**414,914**	**408,903**	**394,181**	**395,960**	**398,505**	**NA**	**NA**	**NA**	**NA**

**Abbreviation:** NA = not applicable.

* Data from 34 reporting areas; excludes 18 areas (California, Connecticut, Delaware, District of Columbia, Florida, Illinois, Maine, Maryland, Massachusetts, Mississippi, Nebraska, New Hampshire, New York State, Pennsylvania, Rhode Island, Vermont, Wisconsin, and Wyoming) that did not report, did not report by weeks of gestation, or did not meet reporting standards for ≥1 year.

^†^ Percentages for the individual component categories might not add to 100% because of rounding.

^§^ By year, the total number of abortions represents 74.6%–98.2% of all abortions reported to CDC among the areas that met reporting standards for gestational age during 2010–2019.

Among the 47 areas that reported by method type for 2019 and included medical abortion on their reporting form, 49.0% of abortions were surgical abortions at ≤13 weeks’ gestation, 42.3% were early medical abortions (a nonsurgical abortion at ≤9 weeks’ gestation), 7.2% were surgical abortions at >13 weeks’ gestation, and 1.4% were medical abortions at >9 weeks’ gestation; other methods, including intrauterine instillation and hysterectomy/hysterotomy, were both uncommon (<0.1%) (Table 12). During 2010−2019, 35 reporting areas (excludes Alabama, Arizona, California, Delaware, District of Columbia, Florida, Hawaii, Illinois, Louisiana, Maine, Maryland, New Hampshire, New Mexico, Tennessee, Vermont, Wisconsin, and Wyoming) provided continuous data and included medical abortion on their reporting form. Among these 35 areas, use of early medical abortion increased 10% from 2018 to 2019 (from 37.5% of abortions to 41.1%) and 123% from 2010 to 2019 (from 18.4% of abortions to 41.1%). 

**TABLE 12:** Number of reported abortions, by known method type and reporting area of occurrence — selected reporting areas,* United States, 2019

State/Area	Surgical^†^			Medical			Intrauterine instillation^§^	Hysterectomy/ Hysterotomy	Total abortions reported by known method type
	Surgical, ≤13 weeks’ gestation	Surgical, >13 weeks’ gestation	Surgical, unknown gestational age	Medical, ≤9 weeks’ gestation	Medical, >9 weeks’ gestation	Medical, unknown gestational age			
	No. (%)^¶^	No. (%)	No. (%)	No. (%)	No. (%)	No. (%)	No. (%)	No. (%)	No. (% of all reported abortions)**
Alabama^††^	3,257 (54.2)	653 (10.9)	—^§§^	2,030 (33.8)	58 (1.0)	—^§§^	0 (—)	0 (—)	**6,004 (99.9)**
Alaska	895 (70.5)	65 (5.1)	0 (—)	305 (24.0)	—^§§^	—^§§^	—^§§^	0 (—)	**1,269 (99.9)**
Arizona	6,768 (51.7)	992 (7.6)	0 (—)	5,031 (38.4)	159 (1.2)	0 (—)	137 (1.0)	0 (—)	**13,087 (99.9)**
Arkansas^††^	1,369 (46.2)	356 (12.0)	0 (—)	829 (28.0)	408 (13.8)	0 (—)	0 (—)	0 (—)	**2,962 (100.0)**
Colorado	2,989 (35.9)	400 (4.8)	—^§§^	4,819 (57.8)	120 (1.4)	0 (—)	—^§§^	0 (—)	**8,334 (92.6)**
Connecticut	3,747 (41.0)	687 (7.5)	136 (1.5)	4,367 (47.8)	25 (0.3)	173 (1.9)	0 (—)	0 (—)	**9,135 (99.3)**
Delaware	722 (36.0)	101 (5.0)	—^§§^	1,136 (56.6)	46 (2.3)	—^§§^	0 (—)	0 (—)	**2,007 (98.3)**
District of Columbia^¶¶^	2,170 (47.7)	382 (8.4)	0 (—)	NA	NA	2,000 (43.9)	0 (—)	0 (—)	**4,552 (100.0)**
Florida	32,315 (47.1)	2,505 (3.7)	0 (—)	33,428 (48.7)	352 (0.5)	0 (—)	0 (—)	8 (0.0)	**68,608 (95.4)**
Georgia	15,801 (42.8)	2,555 (6.9)	0 (—)	18,240 (49.4)	309 (0.8)	0 (—)	0 (—)	0 (—)	**36,905 (100.0)**
Hawaii	1,030 (51.4)	194 (9.7)	—^§§^	776 (38.8)	—^§§^	0 (—)	0 (—)	0 (—)	**2,002 (100.0)**
Idaho	826 (54.7)	42 (2.8)	10 (0.7)	621 (41.1)	8 (0.5)	—^§§^	—^§§^	0 (—)	**1,511 (99.9)**
Indiana	4,241 (55.5)	36 (0.5)	0 (—)	3,297 (43.2)	62 (0.8)	0 (—)	0 (—)	0 (—)	**7,636 (100.0)**
Iowa	948 (26.8)	190 (5.4)	0 (—)	2,364 (66.7)	40 (1.1)	0 (—)	0 (—)	0 (—)	**3,542 (99.3)**
Kansas	1,959 (28.4)	486 (7.1)	0 (—)	4,364 (63.3)	82 (1.2)	0 (—)	0 (—)	0 (—)	**6,891 (100.0)**
Kentucky	1,512 (41.3)	306 (8.4)	0 (—)	1,828 (49.9)	18 (0.5)	0 (—)	0 (—)	0 (—)	**3,664 (100.0)**
Maine	888 (44.0)	106 (5.3)	—^§§^	960 (47.6)	63 (3.1)	—^§§^	0 (—)	0 (—)	**2,018 (99.9)**
Massachusetts***	NA	NA	10,377 (56.4)	NA	NA	7,958 (43.2)	67 (0.4)	0 (—)	**18,402 (99.0)**
Michigan	12,984 (47.6)	2,649 (9.7)	42 (0.2)	11,213 (41.1)	386 (1.4)	10 (0.0)	0 (—)	0 (—)	**27,284 (99.8)**
Minnesota	5,187 (52.2)	958 (9.6)	54 (0.5)	3,589 (36.1)	61 (0.6)	87 (0.9)	—^§§^	—^§§^	**9,940 (100.0)**
Mississippi	725 (22.7)	186 (5.8)	0 (—)	2,228 (69.8)	55 (1.7)	0 (—)	0 (—)	0 (—)	**3,194 (100.0)**
Missouri	1,076 (73.5)	367 (25.1)	0 (—)	5 (0.3)	10 (0.7)	0 (—)	—^§§^	—^§§^	**1,463 (99.5)**
Montana	524 (33.4)	128 (8.2)	0 (—)	900 (57.4)	16 (1.0)	0 (—)	0 (—)	0 (—)	**1,568 (100.0)**
Nebraska	686 (33.2)	122 (5.9)	0 (—)	1,245 (60.2)	13 (0.6)	—^§§^	0 (—)	—^§§^	**2,068 (100.0)**
Nevada	4,599 (55.0)	527 (6.3)	38 (0.5)	3,113 (37.2)	51 (0.6)	37 (0.4)	—^§§^	—^§§^	**8,367 (99.4)**
New Jersey^†††^	12,938 (58.3)	2,692 (12.1)	481 (2.2)	5,896 (26.6)	134 (0.6)	35 (0.2)	0 (—)	0 (—)	**22,176 (100.0)**
New Mexico	1,394 (39.9)	301 (8.6)	58 (1.7)	1,332 (38.2)	338 (9.7)	65 (1.9)	—^§§^	—^§§^	**3,490 (88.5)**
New York	40,495 (52.9)	6,130 (8.0)	1,399 (1.8)	23,809 (31.1)	2,125 (2.8)	2,555 (3.3)	24 (0.0)	32 (0.0)	**76,569 (97.4)**
New York City	29,516 (59.4)	4,113 (8.3)	0 (—)	15,505 (31.2)	525 (1.1)	0 (—)	9 (0.0)	32 (0.1)	**49,700 (99.8)**
New York State	10,979 (40.9)	2,017 (7.5)	1,399 (5.2)	8,304 (30.9)	1,600 (6.0)	2,555 (9.5)	15 (0.1)	0 (—)	**26,869 (93.3)**
North Carolina	12,295 (45.9)	1,992 (7.4)	32 (0.1)	12,209 (45.6)	190 (0.7)	36 (0.1)	0 (—)	12 (0.0)	**26,766 (94.1)**
North Dakota	698 (62.3)	59 (5.3)	—^§§^	361 (32.2)	—^§§^	0 (—)	0 (—)	0 (—)	**1,121 (100.0)**
Ohio	10,350 (51.5)	1,937 (9.6)	—^§§^	7,716 (38.4)	91 (0.5)	0 (—)	0 (—)	—^§§^	**20,097 (100.0)**
Oklahoma	2,152 (43.8)	263 (5.4)	—^§§^	2,460 (50.1)	33 (0.7)	—^§§^	—^§§^	0 (—)	**4,914 (98.4)**
Oregon	3,494 (40.2)	646 (7.4)	21 (0.2)	4,337 (49.9)	133 (1.5)	51 (0.6)	—^§§^	—^§§^	**8,684 (100.0)**
Pennsylvania^§§§^	NA	NA	17,159 (55.3)	NA	NA	13,845 (44.6)	—^§§^	—^§§^	**31,013 (100.0)**
Rhode Island	1,010 (48.3)	179 (8.6)	7 (0.3)	884 (42.2)	12 (0.6)	—^§§^	—^§§^	0 (—)	**2,093 (99.7)**
South Carolina^††^	1,676 (32.9)	319 (6.3)	—^§§^	2,334 (45.8)	766 (15.0)	0 (—)	5 (0.1)	—^§§^	**5,101 (100.0)**
South Dakota	272 (65.7)	0 (—)	—^§§^	124 (30.0)	13 (3.1)	—^§§^	0 (—)	0 (—)	**414 (100.0)**
Tennessee	4,034 (41.5)	716 (7.4)	8 (0.1)	4,765 (49.0)	183 (1.9)	8 (0.1)	0 (—)	5 (0.1)	**9,719 (100.0)**
Texas^††^	30,824 (53.8)	3,906 (6.8)	0 (—)	22,234 (38.8)	305 (0.5)	0 (—)	—^§§^	—^§§^	**57,272 (100.0)**
Utah	1,455 (49.8)	229 (7.8)	0 (—)	1,223 (41.9)	11 (0.4)	0 (—)	—^§§^	—^§§^	**2,921 (100.0)**
Vermont	394 (33.1)	87 (7.3)	—^§§^	683 (57.4)	25 (2.1)	0 (—)	—^§§^	0 (—)	**1,190 (99.6)**
Virginia	9,252 (59.3)	500 (3.2)	15 (0.1)	5,744 (36.8)	74 (0.5)	—^§§^	—^§§^	0 (—)	**15,589 (99.9)**
Washington	7,491 (43.4)	1,317 (7.6)	30 (0.2)	8,320 (48.2)	56 (0.3)	36 (0.2)	0 (—)	0 (—)	**17,250 (99.9)**
West Virginia	617 (52.2)	77 (6.5)	0 (—)	454 (38.4)	35 (3.0)	0 (—)	0 (—)	0 (—)	**1,183 (100.0)**
Wisconsin***^,¶¶¶^	NA	NA	4,207 (66.0)	NA	NA	2,165 (34.0)	0 (—)	0 (—)	**6,372 (100.0)**
Wyoming	0 (—)	0 (—)	—^§§^	30 (96.8)	—^§§^	0 (—)	0 (—)	0 (—)	**31 (100.0)**
**Total**	**277,789 (49.0)**	**40,699 (7.2)**	—****	**239,770 (42.3)**	**7,787 (1.4)**	**—^††††^**	**252 (0.0)**	**81 (0.0)**	**566,378 (98.5)** ^§§§§^

**Abbreviation:** NA = not available.

* Data from 47 reporting areas; excludes five reporting areas (California, Illinois, Louisiana, Maryland, and New Hampshire) that did not report, did not report by method type, or did not meet reporting standards. Areas reporting by method type with unknown gestational age or gestational age reported was not compatible with categorizations presented in this table are included.

^†^ Includes uterine aspiration (might also be called dilation and curettage, aspiration curettage, suction curettage, manual vacuum aspiration, menstrual extraction, sharp curettage) and dilation and evacuation procedures.

^§^ Intrauterine instillations reported at ≤12 weeks’ gestation were considered as unknown for method type.

^¶^ Percentages for the individual component categories might not add to 100% because of rounding.

** Percentage is calculated as the number of abortions reported by known method type divided by the sum of abortions reported by known and unknown method type. Values ≥99.95% are rounded to 100.0%.

^††^ Two weeks were added to the probable postfertilization age to provide a corresponding measure to gestational age based on the clinician’s estimate.

^§§^ Cells with a value in the range of 1–4 or cells that would allow for calculation of these small values have been suppressed.

^¶¶^ Numbers for medical abortions at ≤9 weeks versus >9 weeks are not presented because gestational age reported was not compatible with these categorizations.

*** Numbers for surgical abortions at ≤13 weeks versus >13 weeks and for medical abortions at ≤9 weeks versus >9 weeks are not presented because gestational age data were not provided by method type.

^†††^ Reporting to the central health agency is not required. Data are requested from hospitals and licensed ambulatory care facilities only.

^§§§^ Numbers for surgical abortions ≤13 weeks and >13 weeks and medical abortions ≤9 weeks versus >9 weeks are not presented as gestational age reported was not compatible with these categorizations.

^¶¶¶^ Includes residents only. Wisconsin reports as surgical, unspecified and does not differentiate surgical abortions from hysterectomy/hysterotomy. All abortions were reported as surgical or chemically induced. For this report, all surgical abortions were classified as surgical and all chemical abortions as medical.

**** For the total only, surgical abortions reported without a gestational age were distributed among the surgical abortion categories according to the distribution of surgical abortions at known gestational age.

^††††^ For the total only, medical abortions reported without a gestational age were distributed among the medical abortion categories according to the distribution of medical abortions at known gestational age.

^§§§§^ Percentage based on a total of 575,098 abortions reported among the areas that met reporting standards for method type.

Among the 42 areas that reported abortions categorized by individual weeks of gestation and method type for 2019, surgical abortion accounted for the largest percentage of abortions within every gestational age category, except ≤6 weeks’ gestation (Table 13). At ≤6 weeks’ gestation, surgical abortion accounted for 41.3% of abortions. Surgical abortion accounted for 52.2% of abortions at 7–9 weeks’ gestation, 93.2% of abortions at 10–13 weeks’ gestation, 96.9%–99.2% of abortions at 14–20 weeks’ gestation, and 87.0% of abortions at ≥21 weeks’ gestation. In contrast, medical abortion accounted for 58.6% of abortions at ≤6 weeks’ gestation, 47.8% of abortions at 7–9 weeks’ gestation, 6.8% of abortions at 10–13 weeks’ gestation, 0.8%–2.3% of abortions at 14–20 weeks’ gestation, and 11.6% of abortions at ≥21 weeks’ gestation. For each gestational age category as applicable, abortions performed by intrauterine instillation or hysterectomy/hysterotomy were rare (<0.1%–1.3% of abortions).

**TABLE 13:** Number of reported abortions, by known weeks of gestation and method type — selected reporting areas,* United States, 2019

Method type	Weeks of gestation							Total
	≤6	7–9	10–13	14–15	16–17	18–20	≥21	
	No. (%)^†^	No. (%)	No. (%)	No. (%)	No. (%)	No. (%)	No. (%)	No. (%)
**Surgical^§^**								
≤13 weeks’ gestation	84,850 (41.3)	90,714 (52.2)	59,346 (93.2)	NA	NA	NA	NA	**234,910 (49.2)**
>13 weeks’ gestation	NA	NA	NA	13,965 (99.2)	8,199 (98.3)	7,686 (96.9)	4,094 (87.0)	**33,944 (7.1)**
**Medical^¶^**								
≤9 weeks’ gestation	120,333 (58.6)	82,966 (47.8)	NA	NA	NA	NA	NA	**203,299 (42.6)**
>9 weeks’ gestation	NA	NA	4,339 (6.8)	109 (0.8)	94 (1.1)	185 (2.3)	545 (11.6)	**5,272 (1.1)**
**Intrauterine instillation**	—**	—**	1 (0.0)	2 (0.0)	48 (0.6)	58 (0.7)	60 (1.3)	**169 (0.0)**
**Hysterectomy/ Hysterotomy**	19 (0.0)	28 (0.0)	5 (0.0)	4 (0.0)	3 (0.0)	6 (0.1)	7 (0.1)	**72 (0.0)**
**Total**	**205,202 (100.0)**	**173,708 (100.0)**	**63,691 (100.0)**	**14,080 (100.0)**	**8,344 (100.0)**	**7,935 (100.0)**	**4,706 (100.0)**	**477,666 (100.0)**

**Abbreviation**: NA = not applicable.

* Data from 42 reporting areas; excludes 10 reporting areas (California, District of Columbia, Illinois, Louisiana, Maryland, Massachusetts, New Hampshire, New York State, Pennsylvania, and Wisconsin) that did not report, did not report by weeks of gestation, did not meet reporting standards, or did not have medical abortion as a specific category on their reporting form.

^†^ For each gestational age category, percentages of all method types might not add to 100% because of rounding.

^§^ Includes uterine aspiration (might also be called dilation and curettage, aspiration curettage, suction curettage, manual vacuum aspiration, menstrual extraction, sharp curettage) and dilation and evacuation procedures.

^¶^ The administration of medication or medications to induce an abortion; at ≤9 weeks’ gestation, typically involves the use of mifepristone and misoprostol, and at >9 weeks’ gestation, typically involves the use of vaginal prostaglandins.

** Intrauterine instillations reported at ≤12 weeks’ gestation have not been included with known values.

### Weeks of Gestation by Age Group and Race/Ethnicity

In selected reporting areas, abortions that were categorized by weeks of gestation were further categorized by age and by race/ethnicity (Table 14). In every subgroup for these characteristics, the largest percentage of abortions occurred at ≤9 weeks’ gestation. In 43 reporting areas, by age, 61.3% of adolescents aged <15 years and 73.6% of adolescents aged 15–19 years obtained an abortion at ≤9 weeks’ gestation, compared with ≥78.6% among women in older age groups. Conversely, 19.8% of adolescents aged <15 years and 9.6% of adolescents aged 15–19 years obtained an abortion after 13 weeks’ gestation, compared with 6.8%–7.5% for women in older age groups. In 29 reporting areas, by race/ethnicity, 76.2% of non-Hispanic Black women obtained an abortion at ≤9 weeks’ gestation, compared with 80.6%–82.4% of women in other racial/ethnic groups. Differences in abortions after 13 weeks’ gestation across race/ethnicity were minimal (7.8% among non-Hispanic Black women, compared with 6.1%–7.7% among women in other racial/ethnic groups).

**TABLE 14:** Number of reported abortions, by known weeks of gestation, age group, and race/ethnicity — selected reporting areas, United States, 2019

Characteristic	Weeks of gestation							Total
	≤6	7–9	10–13	14–15	16–17	18–20	≥21	
	No. (%)	No. (%)	No. (%)	No. (%)	No. (%)	No. (%)	No. (%)	No. (%)
Age group (yrs)*^,†^								
<15	306 (27.2)	384 (34.1)	214 (19.0)	73 (6.5)	53 (4.7)	47 (4.2)	50 (4.4)	**1,127 (100.0)**
15–19	14,886 (35.7)	15,785 (37.9)	7,007 (16.8)	1,536 (3.7)	948 (2.3)	962 (2.3)	561 (1.3)	**41,685 (100.0)**
20–24	56,003 (41.3)	50,599 (37.3)	19,052 (14.1)	4,179 (3.1)	2,398 (1.8)	2,142 (1.6)	1,226 (0.9)	**135,599 (100.0)**
25–29	61,791 (43.3)	52,298 (36.7)	18,882 (13.2)	3,925 (2.8)	2,294 (1.6)	2,189 (1.5)	1,242 (0.9)	**142,621 (100.0)**
30–34	43,257 (45.1)	34,165 (35.6)	11,747 (12.3)	2,654 (2.8)	1,523 (1.6)	1,473 (1.5)	1,022 (1.1)	**95,841 (100.0)**
35–39	24,816 (46.1)	18,652 (34.7)	6,425 (11.9)	1,501 (2.8)	960 (1.8)	880 (1.6)	588 (1.1)	**53,822 (100.0)**
≥40	9,053 (49.4)	5,919 (32.3)	1,990 (10.9)	523 (2.9)	344 (1.9)	334 (1.8)	165 (0.9)	**18,328 (100.0)**
**Total**	**210,112 (43.0)**	**177,802 (36.4)**	**65,317 (13.4)**	**14,391 (2.9)**	**8,520 (1.7)**	**8,027 (1.6)**	**4,854 (1.0)**	**489,023 (100.0)**
Race/Ethnicity*^,§^								
Non-Hispanic								
White	51,748 (45.4)	40,580 (35.6)	14,205 (12.5)	2,978 (2.6)	1,741 (1.5)	1,767 (1.6)	963 (0.8)	**113,982 (100.0)**
Black	49,644 (38.1)	49,659 (38.1)	20,818 (16.0)	4,464 (3.4)	2,543 (2.0)	2,190 (1.7)	903 (0.7)	**130,221 (100.0)**
Other	11,606 (47.6)	8,047 (33.0)	2,861 (11.7)	697 (2.9)	409 (1.7)	477 (2.0)	279 (1.1)	**24,376 (100.0)**
Hispanic	35,358 (49.5)	23,471 (32.9)	8,211 (11.5)	1,897 (2.7)	1,020 (1.4)	962 (1.3)	499 (0.7)	**71,418 (100.0)**
**Total**	**148,356 (43.6)**	**121,757 (35.8)**	**46,095 (13.6)**	**10,036 (3.0)**	**5,713 (1.7)**	**5,396 (1.6)**	**2,644 (0.8)**	**339,997 (100.0)**

* Percentages for the individual component categories might not add to 100% because of rounding.

^†^ Data from 43 reporting areas; excludes nine reporting areas (California, District of Columbia, Illinois, Maryland, Massachusetts, New Hampshire, New York State, Pennsylvania, and Wisconsin) that did not report, did not report weeks of gestation by age, or did not meet reporting standards.

^§^ Data from 29 reporting areas; excludes 23 reporting areas (California, Colorado, District of Columbia, Hawaii, Illinois, Iowa, Louisiana, Maine, Maryland, Massachusetts, Missouri, Nebraska, New Hampshire, New York City, New York State, North Dakota, Ohio, Oklahoma, Oregon, Pennsylvania, Rhode Island, Washington, and Wisconsin) that did not report, did not report weeks of gestation by race/ethnicity, or did not meet reporting standards.

### Abortion Mortality

Using national PMSS data (*37*), CDC identified two abortion-related deaths for 2018, the most recent year for which data were reviewed for abortion-related deaths (Table 15). Investigation of these cases indicated that both deaths were related to legal abortion.

**TABLE 15:** Number of deaths and case-fatality rates* for abortion-related deaths reported to CDC, by type of abortion — United States, 1973–2018^†^

Year	Type of abortion				CFR per 100,000 legal abortions
	Induced		Unknown**	Total	
	Legal^§^	Illegal^¶^			
**1973–1977**					**2.09**
1973	25	19	3	**47**	
1974	26	6	1	**33**	
1975	29	4	1	**34**	
1976	11	2	1	**14**	
1977	17	4	0	**21**	
**1978–1982**					**0.78**
1978	9	7	0	**16**	
1979	22	0	0	**22**	
1980	9	1	2	**12**	
1981	8	1	0	**9**	
1982	11	1	0	**12**	
**1983–1987**					**0.66**
1983	11	1	0	**12**	
1984	12	0	0	**12**	
1985	11	1	1	**13**	
1986	11	0	2	**13**	
1987	7	2	0	**9**	
**1988–1992**					**0.74**
1988	16	0	0	**16**	
1989	12	1	0	**13**	
1990	9	0	0	**9**	
1991	11	1	0	**12**	
1992	10	0	0	**10**	
**1993–1997**					**0.52**
1993	6	1	2	**9**	
1994	10	2	0	**12**	
1995	4	0	0	**4**	
1996	9	0	0	**9**	
1997	7	0	0	**7**	
**1998–2002**					**0.63**
1998	9	0	0	**9**	
1999	4	0	0	**4**	
2000	11	0	0	**11**	
2001	7	1	0	**8**	
2002	10	0	0	**10**	
**2003–2007**					**0.60**
2003	10	0	0	**10**	
2004	7	1	0	**8**	
2005	7	0	0	**7**	
2006	7	0	0	**7**	
2007	6	0	0	**6**	
**2008–2012**					**0.65**
2008	12	0	0	**12**	
2009	8	0	0	**8**	
2010	10	0	0	**10**	
2011	2	0	0	**2**	
2012	4	0	0	**4**	
**2013–2018**					**0.41**
2013	4	0	0	**4**	
2014	6	0	0	**6**	
2015	2	0	1	**3**	
2016	6	1	1	**8**	
2017	2	0	0	**2**	
2018	2	0	0	**2**	

**Abbreviation:** CFR = case-fatality rate.

* Number of legal induced abortion-related deaths per 100,000 reported legal induced abortions. Because a substantial number of legal induced abortions occurred outside reporting areas that provided data to CDC, national CFRs (i.e., number of legal induced abortion-related deaths per 100,000 reported legal induced abortions in the United States) were calculated with denominator data from the Guttmacher Institute’s national survey of abortion-providing facilities; for 2018, the CFR was calculated using denominator data for 2017, the most recent year for which data are available. Case-fatality rates were computed for consecutive 5-year periods during 1973–2012 and then for a consecutive 6-year period during 2013–2018 because rates based on <20 cases might be unstable.

^†^ Certain numbers might differ from those in reports published previously because additional information has been supplied to CDC subsequent to publication.

^§^ An abortion is defined as legal if it was performed by a licensed clinician within the limits of state law.

^¶^ An abortion is defined as illegal if it was performed by any person other than a licensed clinician.

** Unknown whether abortion was induced or spontaneous.

The annual number of deaths related to legal induced abortion has fluctuated from year to year since 1973 (Table 15). Because of this variability and the limited number of deaths related to legal induced abortions every year, national case-fatality rates for legal abortion were calculated for consecutive 5-year periods during 1973–2012 and then for a consecutive 6-year period during 2013–2018. The national case-fatality rate for legal induced abortion for 2013–2018 was 0.41 deaths related to legal induced abortions per 100,000 reported legal abortions. This case-fatality rate was lower than the rates for the previous 5-year periods.

## Discussion

For 2019, a total of 629,898 abortions were reported to CDC by 49 areas. Of these reporting areas, 48 submitted data every year for 2010–2019, thus providing the information necessary for consistently reporting trends. Among these 48 areas, for 2019, the abortion rate was 11.4 abortions per 1,000 women aged 15–44 years, and the abortion ratio was 195 abortions per 1,000 live births. From 2018 to 2019, the number of abortions increased 2%, the abortion rate increased 0.9%, and the abortion ratio increased 3%. Although the rate of reported abortions declined overall from 2010 to 2019, after reaching a historic low in 2017, the abortion rates increased overall between 2017 and 2019.

Approximately 18% of all pregnancies in the United States end in induced abortion (*6*). Multiple factors influence the incidence of abortion, including access to health care services and contraception (*40*–*43*); the availability of abortion providers (*4*,*6*,*44*–*47*); state regulations, such as mandatory waiting periods (*48*–*50*), parental involvement laws (*51*,*52*), and legal restrictions on abortion providers (*53*–*57*); and changes in the economy and the resulting impact on family planning decisions and contraceptive use (*58*).

Among areas that reported data continuously by age during 2010–2019, women in their 20s accounted for the majority of abortions and had the highest abortion rates, whereas adolescents aged <15 years had the lowest abortion rates, and adolescents aged <15 years and 15−19 years had the highest abortion ratios. During 2010–2019, women aged ≥40 years accounted for a relatively small proportion of reported abortions (3.4%–3.7%). However, the abortion ratio among women aged ≥40 years continues to be higher than among women aged 25–39 years. These data underscore important age differences in abortion measures.

The trends in adolescent abortions described in this report are important for monitoring trends in adolescent pregnancies in the United States. From 2010 to 2019, national birth data indicate that the birth rate for adolescents aged 15–19 years decreased 51% (*30*), and the data in this report indicate that the abortion rate for the same age group decreased 50%. These findings highlight that decreases in adolescent births in the United States have been accompanied by large decreases in adolescent abortions (*30*).

As in previous years, abortion rates and ratios differ across racial/ethnic groups. For example, in 2019, compared with non-Hispanic White women, abortion rates and ratios were 3.6 and 3.3 times higher among non-Hispanic Black women and 1.8 and 1.5 times higher among Hispanic women. Similar differences have been demonstrated in other U.S.-based studies (*2*,*7*–*10*,*59*). The factors leading to higher abortion rates among certain racial/ethnic minority groups are complex. In addition to disparities in rates of unintended pregnancies, structural factors, including unequal access to quality family planning services (*60*,*61*), economic disadvantage, and distrust of the medical system (*62*), might contribute to observed differences. Strategies are needed to address these broader structural inequities.

In 2019, the majority of abortions occurred early in gestation (≤9 weeks), when the risks for complications are lowest (*63*–*66*). In addition, over the past 10 years, approximately three fourths of abortions were performed at ≤9 weeks’ gestation, and this percentage increased from 74.8% in 2010 to 77.4% in 2019. Moreover, among areas that reported abortions at ≤13 weeks’ gestation by individual week, the distribution of abortions by gestational age continued to shift toward earlier weeks of gestation, with the percentage of early abortions performed at ≤6 weeks’ gestation increasing from 34.7% in 2010 to 37.5% in 2019. Previous research indicates that the distribution of abortions by gestational age differs by various sociodemographic characteristics (*67*–*69*). In this report, the percentage of adolescents aged ≤19 years who obtained abortions at >13 weeks’ gestation was higher than the percentage of abortions obtained among older age groups. The gestational age when abortions are performed might be influenced by multiple factors, including state abortion restrictions, accurate estimation of gestational age, income level, age, and presence of pregnancy-related health conditions (*48*,*59*,*66*,*68*–*73*).

Changes in abortion practices have facilitated the trend of obtaining abortions earlier in pregnancy. Research conducted in the United States during the 1970s indicated that surgical abortion procedures performed at ≤6 weeks’ gestation, compared with 7–12 weeks’ gestation, were less likely to result in successful termination of the pregnancy (*74*). However, subsequent advances in technology (e.g., improved transvaginal ultrasonography and sensitivity of pregnancy tests) have allowed very early surgical abortions to be performed with completion rates exceeding 97% (*75*–*78*). Likewise, the development of early medical abortion regimens has allowed for abortions to be performed early in gestation, with completion rates for regimens that combine mifepristone and misoprostol reaching 96%–98% (*78*–*81*). Among those that were eligible (≤9 weeks’ gestation), 53.7% were reported as early medical abortions. Moreover, among areas that reported by method type and included medical abortion on their reporting form, the percentage of all abortions performed by early medical abortion increased 123% from 2010 to 2019.

Because the annual number of deaths related to legal induced abortion is small and statistically unstable, case-fatality rates were calculated for consecutive 5-year periods during 1973–2012 and then for a consecutive 6-year period during 2013–2018. The national case-fatality rate for legal induced abortion for 2013–2018 was 0.41 deaths per 100,000 abortions; since the late 1970s, all rates for the preceding 5-year periods have been fewer than 1 death per 100,000 abortions, demonstrating the low risk for death associated with legal induced abortion.

## Limitations

The findings in this report are subject to at least four limitations. First, because reporting to CDC is voluntary and reporting requirements vary by the individual reporting areas (*13*), CDC is unable to report the total number of abortions performed in the United States. Of the 52 areas from which CDC requested data for 2019, California, Maryland, and New Hampshire did not submit abortion data. In 2017, the most recent year for which data are available through the Guttmacher Institute’s national survey of abortion-providing facilities, abortions performed in these states accounted for approximately 19% of all abortions in the United States (*6*). In addition, New Jersey did not have abortion reporting requirements to a centralized health agency during the period covered in this report (*12*), which potentially affects the representativeness of data provided to CDC. Some reporting areas (the District of Columbia and Wyoming) implemented new legislation that could improve reporting of 2019 abortion data. Nonetheless, even in reporting areas that legally require clinicians to submit a report for every abortion they perform, enforcement of this requirement varies.

Second, many states use abortion reporting forms that differ from the technical guidance that CDC developed in collaboration with the National Association for Public Health Statistics and Information Systems. Consequently, some reporting areas do not collect all variables requested by CDC (e.g., age and race/ethnicity) or do not report the data in a manner consistent with this guidance (e.g., gestational age). Missing demographic information can reduce the extent to which the statistics in this report represent women who have had abortions. Findings in this report on the age of women seeking abortions were generally similar to previously published data from Guttmacher Institute’s national survey of abortion patients in 2014, although the percentage of abortions among non-Hispanic Black women was lower and among Hispanic women was higher compared with data provided to CDC (*82*). Differences might be attributable to the fact that only 30 reporting areas reported race/ethnicity data to CDC that met CDC’s reporting standards. Some areas that either do not report to CDC (e.g., California) or do not report race/ethnicity data (e.g., Illinois) have sufficiently large populations of racial/ethnic minority groups that the absence of data from these areas likely reduces the representativeness of CDC data for these variables. In addition, some areas collect gestational age data that are based on estimated date of conception or probable postfertilization age, which are not consistent with medical conventions for gestational age reporting. Without medical guidance on how to report these data, the validity and reliability of gestational age for these reporting areas is uncertain.

Third, abortion data are compiled and reported to CDC by the central health agency of the reporting area in which the abortion was performed rather than the reporting area in which the person lived. Thus, the available population (*19*–*28*) and birth data (*29*,*30*), which are organized by the states in which women live, might differ from the population of women who undergo abortions in a given reporting area. This likely results in an overestimation of abortions for reporting areas in which a higher percentage of abortions are obtained by out-of-state residents and an underestimation of abortions for reporting areas where residents more frequently obtain abortions out of state. Limited abortion services, stringent regulatory requirements for obtaining an abortion, or geographic proximity to services in another state might influence where women obtain abortion services (*83*).

Finally, CDC reporting of sociodemographic characteristics of women obtaining abortions is limited to data collected on jurisdiction reporting forms. Therefore, examining additional demographic variables (e.g., socioeconomic status) is not possible.

## Public Health Implications

Ongoing surveillance of legal induced abortion is important for several reasons. First, abortion surveillance can be used to help evaluate programs aimed at promoting equitable access to patient-centered contraceptive care in the United States to reduce unintended pregnancies. Cost, inadequate provider reimbursement and training, insufficient patient-centered counseling, lack of youth-friendly services, and low client awareness of available contraceptive methods are reported barriers to accessing contraception (*40*–*42*,*84*–*90*). Reducing these barriers might help ensure equitable access to patient-centered contraceptive care and promote equitable reproductive health in the United States (*91*).

Second, routine abortion surveillance is needed to assess trends in clinical practice patterns over time. Information in this report on the number of abortions performed through different methods (e.g., medical or surgical) and at different gestational ages provides the denominator data that are necessary for analyses of the relative safety of abortion practices (*38*). Finally, information on the number of pregnancies ending in abortion is needed in conjunction with data on births and fetal losses to estimate the number of pregnancies in the United States and determine rates for various outcomes of public health importance (e.g., adolescent pregnancies) (*11*).

## References

[1] Gamble SB, Strauss LT, Parker WY, Cook DA, Zane SB, Hamdan S; CDC. Abortion surveillance—United States, 2005. MMWR Surveill Summ 2008;57(No. SS-13). 19037196

[2] Jones RK, Kost K, Singh S, Henshaw SK, Finer LB. Trends in abortion in the United States. Clin Obstet Gynecol 2009;52:119–29. 10.1097/GRF.0b013e3181a2af8f 19407518

[3] Pazol K, Zane SB, Parker WY, Hall LR, Berg C, Cook DA; CDC. Abortion surveillance—United States, 2008. MMWR Surveill Summ 2011;60(No. SS-15). 22108620

[4] Jones RK, Kooistra K. Abortion incidence and access to services in the United States, 2008. Perspect Sex Reprod Health 2011;43:41–50. 10.1363/4304111 21388504

[5] Kortsmit K, Jatlaoui TC, Mandel MG, Abortion surveillance—United States, 2018. MMWR Surveill Summ 2020;69(No. SS-7). 10.15585/mmwr.ss6907a1 33237897 PMC7713711

[6] Jones RK, Witwer E, Jerman J. Abortion incidence and service availability in the United States, 2017. New York, NY: Guttmacher Institute; 2019. https://www.guttmacher.org/report/abortion-incidence-service-availability-us-2017

[7] Henshaw SK, Silverman J. The characteristics and prior contraceptive use of U.S. abortion patients. Fam Plann Perspect 1988;20:158–68. 10.2307/2135791 3243346

[8] Jones RK, Darroch JE, Henshaw SK. Patterns in the socioeconomic characteristics of women obtaining abortions in 2000–2001. Perspect Sex Reprod Health 2002;34:226–35. 10.2307/3097821 12392215

[9] Jones RK, Kavanaugh ML. Changes in abortion rates between 2000 and 2008 and lifetime incidence of abortion. Obstet Gynecol 2011;117:1358–66. 10.1097/AOG.0b013e31821c405e 21606746

[10] Jones RK, Jerman J. Population group abortion rates and lifetime incidence of abortion: United States, 2008–2014. Am J Public Health 2017;107:1904–9. 10.2105/AJPH.2017.304042 29048970 PMC5678377

[11] Maddow-Zimet I, Kost K. Pregnancies, births and abortions in the United States, 1973–2017: national and state trends by age. New York, NY: Guttmacher Institute; 2021. https://www.guttmacher.org/report/pregnancies-births-abortions-in-united-states-1973-2017

[12] Guttmacher Institute. Abortion reporting requirements. New York, NY: Guttmacher Institute; 2021. https://www.guttmacher.org/state-policy/explore/abortion-reporting-requirements

[13] Saul R. Abortion reporting in the United States: an examination of the federal-state partnership. Fam Plann Perspect 1998;30:244–7. 10.2307/2991612 9782049

[14] National Center for Health Statistics. Guide to completing the facility worksheets for the certificate of live birth and report of fetal death. Hyattsville, MD: US Department of Health and Human Services, CDC, National Center for Health Statistics; 2016. https://www.cdc.gov/nchs/data/dvs/GuidetoCompleteFacilityWks.pdf

[15] Fritz MA, Speroff L. Clinical gynecologic endocrinology and infertility. Philadelphia, PA: Wolters Kluwer Health; 2012.

[16] Mifeprex (mifepristone) [Package insert]. New York, NY: Danco Laboratories; 2016.

[17] Paul M, Lichtenberg ES, Borgatta L, Grimes DA, Stubblefield PG, Creinin MD. Management of unintended and abnormal pregnancy: comprehensive abortion care. Oxford, England: Blackwell Publishing Ltd.; 2009.

[18] Lichtman AS, Brenner P, Mishell DR Jr. Intrauterine administration of prostaglandin F2alpha as an outpatient procedure for termination of early pregnancy. Contraception 1974;9:403–8. 10.1016/0010-7824(74)90083-3 4442283

[19] CDC. Bridged-race population estimates, April 1, 2010. [File census_0401_2010.sas7bdat.zip]. Hyattsville, MD: US Department of Health and Human Services, CDC, National Center for Health Statistics; 2011. https://www.cdc.gov/nchs/nvss/bridged_race/data_documentation.htm#april2010

[20] CDC. Postcensal estimates of the resident population of the United States as of July 1, 2011, by year, state and county, age, bridged race, sex, and Hispanic origin (Vintage 2011). [File pcen_v2011_y11.sas7bdat]. Hyattsville, MD: US Department of Health and Human Services, CDC, National Center for Health Statistics; 2012. https://www.cdc.gov/nchs/nvss/bridged_race/data_documentation.htm#vintage2011

[21] CDC. Postcensal estimates of the resident population of the United States as of July 1, 2012, by year, state and county, age, bridged race, sex, and Hispanic origin (Vintage 2012) [File pcen_v2012_y12.sas7bdat]. Hyattsville, MD: US Department of Health and Human Services, CDC, National Center for Health Statistics; 2013. https://www.cdc.gov/nchs/nvss/bridged_race/data_documentation.htm#vintage2012

[22] CDC. Postcensal estimates of the resident population of the United States as of July 1, 2013, by year, state and county, age, bridged race, sex, and Hispanic origin (Vintage 2013) [File pcen_v2013_y13.sas7bdat]. Hyattsville, MD: US Department of Health and Human Services, CDC, National Center for Health Statistics; 2014. https://www.cdc.gov/nchs/nvss/bridged_race/data_documentation.htm#vintage2013

[23] CDC. Postcensal estimates of the resident population of the United States as of July 1, 2014, by year, state and county, age, bridged race, sex, and Hispanic origin (Vintage 2014). [File pcen_v2014_y14.sas7bdat]. Hyattsville, MD: US Department of Health and Human Services, CDC, National Center for Health Statistics; 2015. http://www.cdc.gov/nchs/nvss/bridged_race/data_documentation.htm#vintage2014

[24] CDC. Postcensal estimates of the resident population of the United States as of July 1, 2015, by year, state and county, age, bridged race, sex, and Hispanic origin (Vintage 2015). [File pcen_v2015_y15.sas7bdat]. Hyattsville, MD: US Department of Health and Human Services, CDC, National Center for Health Statistics; 2016. https://www.cdc.gov/nchs/nvss/bridged_race/data_documentation.htm#vintage2015

[25] CDC. Postcensal estimates of the resident population of the United States as of July 1, 2016, by year, state and county, age, bridged race, sex, and Hispanic origin (Vintage 2016). [File pcen_v2016_y16.sas7bdat]. Hyattsville, MD: US Department of Health and Human Services, CDC, National Center for Health Statistics; 2017. https://www.cdc.gov/nchs/nvss/bridged_race/data_documentation.htm#Vintage2016

[26] CDC. Postcensal estimates of the resident population of the United States as of July 1, 2017, by year, state and county, age, bridged race, sex, and Hispanic origin (Vintage 2017). [File pcen_v2017_y17.sas7bdat]. Hyattsville, MD: US Department of Health and Human Services, CDC, National Center for Health Statistics; 2018. https://www.cdc.gov/nchs/nvss/bridged_race/data_documentation.htm#Vintage2017

[27] CDC. Postcensal estimates of the resident population of the United States as of July 1, 2018, by year, state and county, age, bridged race, sex, and Hispanic origin (Vintage 2018). [File pcen_v2018_y18.sas7bdat]. Hyattsville, MD: US Department of Health and Human Services, CDC, National Center for Health Statistics; 2019. https://www.cdc.gov/nchs/nvss/bridged_race/data_documentation.htm#Vintage2018

[28] CDC. Postcensal estimates of the resident population of the United States as of July 1, 2019, by year, state and county, age, bridged race, sex, and Hispanic origin (Vintage 2019). [File pcen_v2019_y19.sas7bdat]. Hyattsville, MD: US Department of Health and Human Services, CDC, National Center for Health Statistics; 2020. https://www.cdc.gov/nchs/nvss/bridged_race/data_documentation.htm#Vintage2019

[29] CDC. Natality files. Hyattsville, MD: US Department of Health and Human Services, CDC, National Center for Health Statistics. https://wonder.cdc.gov/Natality.html

[30] Martin JA, Hamilton BE, Osterman MJK, Driscoll AK. Births: final data for 2019. Natl Vital Stat Rep 2021;70:1–51. 33814033

[31] Winikoff B, Dzuba IG, Chong E, Extending outpatient medical abortion services through 70 days of gestational age. Obstet Gynecol 2012;120:1070–6. 10.1097/AOG.0b013e31826c315f 23090524

[32] National Abortion Federation. 2013 clinical policy guidelines. Washington, DC: National Abortion Federation; 2013. https://www.prochoice.org/pubs_research/publications/documents/2013NAFCPGsforweb.pdf

[33] Creinin M, Grossman DA. Medical management of first-trimester abortion. Contraception 2014;89:148–61. 10.1016/j.contraception.2014.01.016 24795934

[34] Food and Drug Administration. Mifeprex (mifepristone) information. Silver Spring, MD: US Department of Health and Human Services, Food and Drug Administration; 2016. https://www.fda.gov/Drugs/DrugSafety/PostmarketDrugSafetyInformationforPatientsandProviders/ucm111323.htm

[35] CDC. Abortion surveillance, 1972. Atlanta, GA: US Department of Health, Education, and Welfare, Public Health Service, CDC; 1974.

[36] CDC. Abortion surveillance, 1977. Atlanta, GA: US Department of Health, Education, and Welfare, Public Health Service, CDC; 1979.

[37] CDC. Pregnancy Mortality Surveillance System. Atlanta, GA: US Department of Health and Human Services, CDC; 2020. https://www.cdc.gov/reproductivehealth/maternal-mortality/pregnancy-mortality-surveillance-system.htm

[38] Zane S, Creanga AA, Berg CJ, Abortion-related mortality in the United States: 1998–2010. Obstet Gynecol 2015;126:258–65. 10.1097/AOG.0000000000000945 26241413 PMC4554338

[39] Hoyert DL. Maternal mortality and related concepts. Vital Health Stat 3 2007;33:1–13. 17460868

[40] Peipert JF, Madden T, Allsworth JE, Secura GM. Preventing unintended pregnancies by providing no-cost contraception. Obstet Gynecol 2012;120:1291–7. 10.1097/AOG.0b013e318273eb56 23168752 PMC4000282

[41] Biggs MA, Rocca CH, Brindis CD, Hirsch H, Grossman D. Did increasing use of highly effective contraception contribute to declining abortions in Iowa? Contraception 2015;91:167–73. 10.1016/j.contraception.2014.10.009 25465890

[42] Ricketts S, Klingler G, Schwalberg R. Game change in Colorado: widespread use of long-acting reversible contraceptives and rapid decline in births among young, low-income women. Perspect Sex Reprod Health 2014;46:125–32. 10.1363/46e1714 24961366

[43] Roth LP, Sanders JN, Simmons RG, Bullock H, Jacobson E, Turok DK. Changes in uptake and cost of long-acting reversible contraceptive devices following the introduction of a new low-cost levonorgestrel IUD in Utah’s Title X clinics: a retrospective review. Contraception 2018;98:63–8. 10.1016/j.contraception.2018.03.029 29574095 PMC6207500

[44] Jones RK, Jerman J. Abortion incidence and service availability in the United States, 2011. Perspect Sex Reprod Health 2014;46:3–14. 10.1363/46e0414 24494995

[45] Finer LB, Henshaw SK. Abortion incidence and services in the United States in 2000. Perspect Sex Reprod Health 2003;35:6–15. 10.1363/3500603 12602752

[46] Henshaw SK. Abortion incidence and services in the United States, 1995–1996. Fam Plann Perspect 1998;30:263–70, 287. 10.2307/2991501 9859016

[47] Quast T, Gonzalez F, Ziemba R. Abortion facility closings and abortion rates in Texas. Inquiry 2017;54:46958017700944. 10.1177/0046958017700944 28351188 PMC5798726

[48] Joyce TJ, Henshaw SK, Dennis A, Finer LB, Blanchard K. The impact of state mandatory counseling and waiting period laws on abortion: a literature review. New York, NY: Guttmacher Institute; 2009. https://www.guttmacher.org/pubs/MandatoryCounseling.pdf

[49] Sanders JN, Conway H, Jacobson J, Torres L, Turok DK. The longest wait: examining the impact of Utah’s 72-hour waiting period for abortion. Womens Health Issues 2016;26:483–7. 10.1016/j.whi.2016.06.004 27502901

[50] Ely G, Polmanteer RSR, Caron A. Access to abortion services in Tennessee: does distance traveled and geographic location influence return for a second appointment as required by the mandatory waiting period policy? Health Soc Work 2019;44:13–21. 10.1093/hsw/hly039 30561624

[51] Dennis A, Henshaw SK, Joyce TJ, Finer LB, Blanchard K. The impact of laws requiring parental involvement for abortion: a literature review. New York, NY: Guttmacher Institute; 2009. https://www.guttmacher.org/pubs/ParentalInvolvementLaws.pdf

[52] Ramesh S, Zimmerman L, Patel A. Impact of parental notification on Illinois minors seeking abortion. J Adolesc Health 2016;58:290–4. 10.1016/j.jadohealth.2015.11.004 26794433

[53] Grossman D, Baum S, Fuentes L, Change in abortion services after implementation of a restrictive law in Texas. Contraception 2014;90:496–501. 10.1016/j.contraception.2014.07.006 25128413 PMC4179978

[54] Joyce T. The supply-side economics of abortion. N Engl J Med 2011;365:1466–9. 10.1056/NEJMp1109889 22010912

[55] Grossman D, White K, Hopkins K, Potter JE. Change in distance to nearest facility and abortion in Texas, 2012 to 2014. JAMA 2017;317:437–9. 10.1001/jama.2016.17026 28114666 PMC8300975

[56] White K, Baum SE, Hopkins K, Potter JE, Grossman D. Change in second-trimester abortion after implementation of a restrictive state law. Obstet Gynecol 2019;133:771–9. 10.1097/AOG.0000000000003183 30870293 PMC6435408

[57] Jones RK, Ingerick M, Jerman J. Differences in abortion service delivery in hostile, middle-ground, and supportive states in 2014. Womens Health Issues 2018;28:212–8. 10.1016/j.whi.2017.12.003 29339010 PMC5959790

[58] Guttmacher Institute. A real-time look at the impact of the recession on women’s family planning and pregnancy decisions. New York, NY: Guttmacher Institute; 2009. https://www.guttmacher.org/pubs/RecessionFP.pdf

[59] Jones RK, Jerman J. Characteristics and circumstances of U.S. women who obtain very early and second-trimester abortions. PLoS One 2017;12:e0169969. 10.1371/journal.pone.0169969 28121999 PMC5266268

[60] Dehlendorf C, Rodriguez MI, Levy K, Borrero S, Steinauer J. Disparities in family planning. Am J Obstet Gynecol 2010;202:214–20. 10.1016/j.ajog.2009.08.022 20207237 PMC2835625

[61] Pazol K, Robbins CL, Black LI, Receipt of selected preventive health services for women and men of reproductive age—United States, 2011–2013. MMWR Surveill Summ 2017;66(No. SS-20). 10.15585/mmwr.ss6620a1 29073129 PMC5879726

[62] Dehlendorf C, Harris LH, Weitz TA. Disparities in abortion rates: a public health approach. Am J Public Health 2013;103:1772–9. 10.2105/AJPH.2013.301339 23948010 PMC3780732

[63] Buehler JW, Schulz KF, Grimes DA, Hogue CJ. The risk of serious complications from induced abortion: do personal characteristics make a difference? Am J Obstet Gynecol 1985;153:14–20. 10.1016/0002-9378(85)90582-4 4036997

[64] Ferris LE, McMain-Klein M, Colodny N, Fellows GF, Lamont J. Factors associated with immediate abortion complications. CMAJ 1996;154:1677–85. 8646655 PMC1487918

[65] Bartlett LA, Berg CJ, Shulman HB, Risk factors for legal induced abortion-related mortality in the United States. Obstet Gynecol 2004;103:729–37. 10.1097/01.AOG.0000116260.81570.60 15051566

[66] Lichtenberg ES, Paul M; Society of Family Planning. Surgical abortion prior to 7 weeks of gestation. Contraception 2013;88:7–17. 10.1016/j.contraception.2013.02.008 23574709

[67] Foster DG, Kimport K. Who seeks abortions at or after 20 weeks? Perspect Sex Reprod Health 2013;45:210–8. 10.1363/4521013 24188634

[68] Jones RK, Finer LB. Who has second-trimester abortions in the United States? Contraception 2012;85:544–51. 10.1016/j.contraception.2011.10.012 22176796

[69] Kiley JW, Yee LM, Niemi CM, Feinglass JM, Simon MA. Delays in request for pregnancy termination: comparison of patients in the first and second trimesters. Contraception 2010;81:446–51. 10.1016/j.contraception.2009.12.021 20399953

[70] Drey EA, Foster DG, Jackson RA, Lee SJ, Cardenas LH, Darney PD. Risk factors associated with presenting for abortion in the second trimester. Obstet Gynecol 2006;107:128–35. 10.1097/01.AOG.0000189095.32382.d0 16394050

[71] Finer LB, Frohwirth LF, Dauphinee LA, Singh S, Moore AM. Timing of steps and reasons for delays in obtaining abortions in the United States. Contraception 2006;74:334–44. 10.1016/j.contraception.2006.04.010 16982236

[72] Goyal V, Wallace R, Dermish AI, Factors associated with abortion at 12 or more weeks gestation after implementation of a restrictive Texas law. Contraception 2020;102:314–7. 10.1016/j.contraception.2020.06.007 32592799 PMC7606493

[73] Janiak E, Kawachi I, Goldberg A, Gottlieb B. Abortion barriers and perceptions of gestational age among women seeking abortion care in the latter half of the second trimester. Contraception 2014;89:322–7. 10.1016/j.contraception.2013.11.009 24332434

[74] Kaunitz AM, Rovira EZ, Grimes DA, Schulz KF. Abortions that fail. Obstet Gynecol 1985;66:533–7. 4047543

[75] Creinin MD, Edwards J. Early abortion: surgical and medical options. Curr Probl Obstet Gynecol Fertil 1997;20:1–32.

[76] Edwards J, Carson SA. New technologies permit safe abortion at less than six weeks’ gestation and provide timely detection of ectopic gestation. Am J Obstet Gynecol 1997;176:1101–6. 10.1016/S0002-9378(97)70410-1 9166176

[77] Paul ME, Mitchell CM, Rogers AJ, Fox MC, Lackie EG. Early surgical abortion: efficacy and safety. Am J Obstet Gynecol 2002;187:407–11. 10.1067/mob.2002.123898 12193934

[78] Baldwin MK, Bednarek PH, Russo J. Safety and effectiveness of medication and aspiration abortion before or during the sixth week of pregnancy: A retrospective multicenter study. Contraception 2020;102:13–7. 10.1016/j.contraception.2020.04.004 32298713

[79] Kapp N, Baldwin MK, Rodriguez MI. Efficacy of medical abortion prior to 6 gestational weeks: a systematic review. Contraception 2018;97:90–9. 10.1016/j.contraception.2017.09.006 28935220

[80] Kapp N, Eckersberger E, Lavelanet A, Rodriguez MI. Medical abortion in the late first trimester: a systematic review. Contraception 2019;99:77–86. 10.1016/j.contraception.2018.11.002 30444970 PMC6367561

[81] Nippita S, Paul M. Abortion. In: Hatcher R, Nelson A, Trussell J, et al., eds. Contraceptive technology. 21st ed. New York, NY: Ayer Company Publishers, Inc.; 2018:779–827.

[82] Jerman J, Jones RK, Onda T. Characteristics of U.S. abortion patients in 2014 and changes since 2008. New York, NY: Guttmacher Institute; 2016. https://www.guttmacher.org/sites/default/files/report_pdf/characteristics-us-abortion-patients-2014.pdf

[83] Jerman J, Frohwirth L, Kavanaugh ML, Blades N. Barriers to abortion care and their consequences for patients traveling for services: qualitative findings from two states. Perspect Sex Reprod Health 2017;49:95–102. 10.1363/psrh.12024 28394463 PMC5953191

[84] Goyal V, Canfield C, Aiken ARA, Dermish A, Potter JE. Postabortion contraceptive use and continuation when long-acting reversible contraception is free. Obstet Gynecol 2017;129:655–62. 10.1097/AOG.0000000000001926 28277358 PMC5364035

[85] Gyllenberg FK, Saloranta TH, But A, Gissler M, Heikinheimo O. Induced abortion in a population entitled to free-of-charge long-acting reversible contraception. Obstet Gynecol 2018;132:1453–60. 10.1097/AOG.0000000000002966 30399102

[86] Biggs MA, Taylor D, Upadhyay UD. Role of insurance coverage in contraceptive use after abortion. Obstet Gynecol 2017;130:1338–46. 10.1097/AOG.0000000000002361 29112661

[87] Boulet SL, D’Angelo DV, Morrow B, Contraceptive use among nonpregnant and postpartum women at risk for unintended pregnancy, and female high school students, in the context of Zika preparedness—United States, 2011–2013 and 2015. MMWR Morb Mortal Wkly Rep 2016;65:780–7. 10.15585/mmwr.mm6530e2 27490117

[88] Kumar N, Brown JD. Access barriers to long-acting reversible contraceptives for adolescents. J Adolesc Health 2016;59:248–53. 10.1016/j.jadohealth.2016.03.039 27247239

[89] Parks C, Peipert JF. Eliminating health disparities in unintended pregnancy with long-acting reversible contraception (LARC). Am J Obstet Gynecol 2016;214:681–8. 10.1016/j.ajog.2016.02.017 26875950 PMC4884485

[90] Klein DA, Berry-Bibee EN, Keglovitz Baker K, Malcolm NM, Rollison JM, Frederiksen BN. Providing quality family planning services to LGBTQIA individuals: a systematic review. Contraception 2018;97:378–91. 10.1016/j.contraception.2017.12.016 29309754

[91] Holt K, Reed R, Crear-Perry J, Scott C, Wulf S, Dehlendorf C. Beyond same-day long-acting reversible contraceptive access: a person-centered framework for advancing high-quality, equitable contraceptive care. Am J Obstet Gynecol 2020;222(4S):878.e1, 878.e6. 10.1016/j.ajog.2019.11.1279 31809706

