# *Erratum:* Vol. 70, No. SS-9

**DOI:** 10.15585/mmwr.mm7139a5

**Published:** 2022-09-30

In the Surveillance Summary “Abortion Surveillance — United States, 2019,” on page 14, in Table 2, under the column heading “Abortions obtained by out-of-state residents,” the value for New York should have read **4,166 (5.3)**.

